# Bromodomain-Containing Protein BRD4 Is Hyperphosphorylated in Mitosis

**DOI:** 10.3390/cancers12061637

**Published:** 2020-06-20

**Authors:** Ranran Wang, June F. Yang, Flora Ho, Erle S. Robertson, Jianxin You

**Affiliations:** 1 Department of Microbiology, Perelman School of Medicine, University of Pennsylvania, Philadelphia, PA 19104, USA; ranran@pennmedicine.upenn.edu (R.W.); june.yang@pennmedicine.upenn.edu (J.F.Y.); florah@sas.upenn.edu (F.H.); 2 Department of Otorhinolaryngology-Head and Neck Surgery, Perelman School of Medicine, University of Pennsylvania, Philadelphia, PA 19104, USA; erle@pennmedicine.upenn.edu

**Keywords:** bromodomain-containing protein 4 (BRD4), protein phosphorylation, BETi resistance, mitosis, cell cycle, Cyclin-dependent kinase 1 (CDK1)

## Abstract

The epigenetic reader BRD4 binds acetylated histones and plays a central role in controlling cellular gene transcription and proliferation. Dysregulation of BRD4′s activity has been implicated in the pathogenesis of a wide variety of cancers. While blocking BRD4 interaction with acetylated histones using BET inhibitors (BETis) has been tested in clinical trials, many cancers have acquired BETi resistance. However, the underlying mechanisms are poorly understood and BETi resistance remains a pressing clinical problem. We previously showed that BRD4 phosphorylation supports stronger chromatin binding and target oncogene expression. In this study, we discovered that BRD4 is hyperphosphorylated by CDK1 during mitosis and determined the major CDK1 phosphorylation sites in BRD4. Using CRISPR/Cas9 gene editing, we replaced endogenous BRD4 with a non-phosphorylatable mutant and demonstrated that CDK1-mediated BRD4 phosphorylation contributes to BETi resistance. CDK1 over-activation frequently observed in cancers has the potential to cause aberrant BRD4 hyperphosphorylation persisting outside of mitosis to strengthen its target gene binding and confer BETi resistance. We found that dual CDK1 and BET inhibition generates a synergistic effect in killing BETi-resistant cancer cells. Our study therefore suggests that CDK1 inhibition can be employed to overcome tumor BETi resistance and improve treatments for BRD4-associated cancers.

## 1. Introduction

Bromodomain-containing protein 4 (BRD4) belongs to the bromodomain and extraterminal (BET) family of proteins. It binds acetylated histones on chromatin through its bromodomains [1], and actively recruits P-TEFb (positive transcription elongation factor b) to facilitate transcriptional activation of RNA polymerase II (RNAPII) [2,3,4]. BRD4 binds M/G1 growth-associated genes during mitosis and functions as a mitotic bookmarker, preserving the epigenetic memory of these genes throughout mitosis to ensure their rapid postmitotic transcriptional re-activation [5,6,7,8]. In addition, we and others have shown that BRD4 function is critical for chromosome segregation in normal cells [9,10]. BRD4 therefore functions as an epigenetic reader and plays a central role in transcriptional regulation and cellular growth control [2,5,6,7,8,10,11,12,13].

Aberrant BRD4 function has been implicated in the pathogenesis of a wide range of cancers [4,14,15,16,17,18,19,20,21,22,23,24,25,26,27]. In cancer cells, BRD4 is disproportionately enriched at certain key oncogenic genes, such as c-MYC, and selectively upregulates their expression to drive cellular proliferation [4,14,16,17,25,28,29]. BRD4 has thus emerged as a critical therapeutic target for a wide variety of cancers [4,14,15,16,17,18,19,20,21,22,23,24,25,27,30]. The BET inhibitors (BETis) JQ1 [31] and I-BET [32], which block BRD4 binding to acetylated histones and specifically downregulate key downstream oncogenes, have been used to treat BRD4-associated cancers [4,25,28]. BETi clinical trials have revealed promising results [32,33], demonstrating the potential of targeting BRD4 in cancer therapy. However, many of the affected cancers have also acquired BETi resistance [34,35,36,37], highlighting the limitations of BETi therapy and the complex nature of BRD4′s regulation. Furthermore, we and others have shown that BRD4 plays an important role in controlling the expression of pluripotency and growth-related genes in noncancerous systems such as mouse embryonic stem cells (mESCs) and preimplantation embryos [12,38,39,40,41]. Hence, there are concerns that BETis can disrupt BRD4 functions in normal cells and may produce unintended consequences [42]. It is therefore imperative to elucidate the molecular mechanisms regulating BRD4 in both normal and disease settings so that novel therapeutic strategies can be developed to specifically inhibit BRD4′s oncogenic functions. However, the cellular pathways that regulate BRD4 function and the mechanistic role of BRD4 alternation in cancer remain largely unexplored.

We recently discovered that BRD4 is hyperphosphorylated in the highly lethal NUT midline carcinoma (NMC) to promote its target oncogene activation and tumor development [43]. We found that, compared to unphosphorylated BRD4 in noncancerous cells, hyperphosphorylated BRD4 observed in NMC binds chromatin with much higher affinity [44], suggesting a mechanism by which BRD4 hyperphosphorylation stimulates abnormal induction of target oncogenes to drive highly aggressive NMC oncogenesis [43,44,45,46]. Since activation of the BRD4-dependent gene expression is a core oncogenic driver for leukemia diseases [14,47,48,49,50,51,52,53] and Burkitt’s lymphoma [17], we also analyzed BRD4′s phosphorylation status in a number of leukemia and lymphoma cell lines [43]. In contrast to noncancerous human dermal fibroblasts (HDFs), which maintain a basal level of BRD4 phosphorylation, various degrees of BRD4 hyperphosphorylation were observed in cancer cells exhibiting elevated BRD4 oncogenic activity [43]. This finding suggests that BRD4 hyperphosphorylation may be a general mechanism contributing to its oncogenic function.

In this study, we discovered that BRD4 is hyperphosphorylated specifically during mitosis in both established cancer cell lines as well as normal HDFs. We demonstrated that CDK1 (Cyclin dependent kinase 1) is largely responsible for BRD4 mitotic hyperphosphorylation. Our studies suggest that CDK1-mediated BRD4 mitotic hyperphosphorylation contributes to its mitotic bookmarking function and that dysregulation of this process could promote BETi resistance. We also provide experimental evidence to support that blocking BRD4 hyperphosphorylation with CDK1 inhibitors could be employed to overcome BETi resistance in cancer.

## 2. Results

### 2.1. BRD4 Is Hyperphosphorylated during Mitosis

Our previous study showed that BRD4 is hyperphosphorylated in NMC cells to support downstream oncogene expression and cellular transformation [43]. To fully understand how BRD4 phosphorylation regulates its biological function, we performed a small molecule screen to identify compounds affecting the phosphorylation status of BRD4. As described previously, BRD4 phosphorylation was monitored using an acrylamide gel containing Phos-tag, which specifically binds to phosphorylated proteins and selectively retards their migration during electrophoresis [54]. Through screening a library of FDA-approved anticancer drugs, we identified seven chemicals, including ixabepilone, vinblastine sulfate, vincristine sulfate, paclitaxel, vinorelbine tartrate, cabazitaxel and docetaxel that could induce BRD4 hyperphosphorylation in HEK293 cells when compared to DMSO-treated control samples (Figure S1).

**Figure 1:**
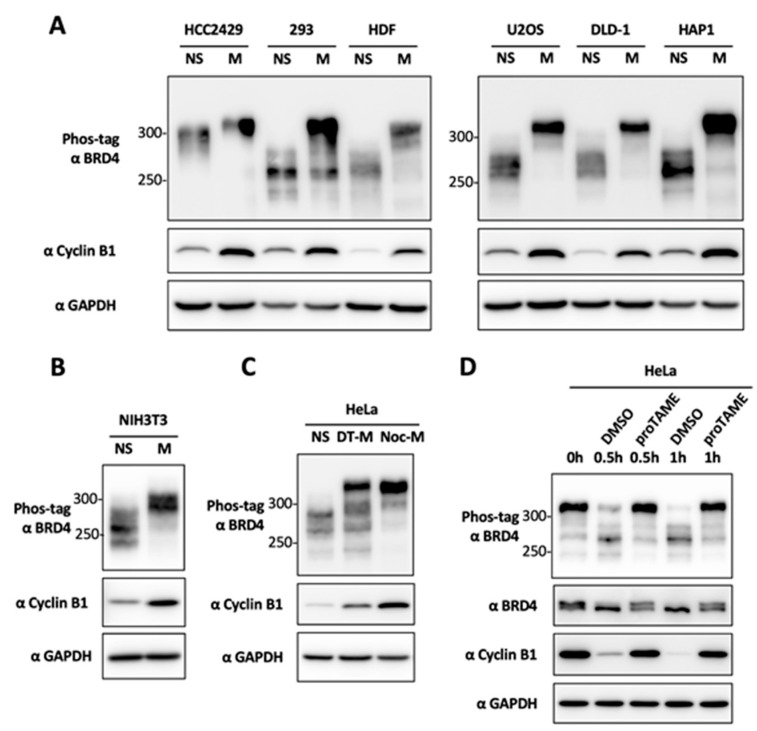
BRD4 is hyperphosphorylated during mitosis. (**A**) HCC2429, HEK293, HDF, U2OS, DLD-1 and HAP1 cells were not synchronized (NS) or synchronized in mitosis (M) using nocodazole. Whole-cell lysates were resolved in Phos-tag or SDS/PAGE gels and immunoblotted using the indicated antibodies. (**B**) Mouse NIH3T3 cells were not synchronized (NS) or synchronized in mitosis (M) using nocodazole and analyzed as in (**A**). (**C**) HeLa cells were not synchronized (NS) or synchronized in mitosis (M) using nocodazole (Noc-M). Mitotic HeLa cells were also collected by shake-off at ten hours after releasing from double thymidine block (DT-M). Whole-cell lysates were analyzed as in (**A**). (**D**) Mitotic HeLa cells collected using mitotic shake-off were treated with DMSO or 12 μM proTAME and harvested at the indicated time points. Whole-cell lysates were analyzed as in (**A**).

Remarkably, the small molecule compounds we found to be capable of inducing BRD4 hyperphosphorylation are all anti-microtubule agents that can disrupt mitotic spindle structure and arrest the cell cycle in mitosis [55,56,57,58]. We therefore speculated that BRD4 is hyperphosphorylated specifically during mitosis. To test this hypothesis, we first treated the cells with nocodazole, another anti-microtubule drug, to arrest cells in mitosis, and then applied mitotic shake off [59] to purify mitotic cells from several types of actively growing cells including NMC HCC2429, HEK293, HDFs, U2Os, DLD-1 and HAP1 cells. As established in our previous studies, BRD4 hyperphosphorylation was not only detected in asynchronous HCC2429 cells, but also slightly increased in the HCC2429 cells arrested in mitosis (Figure 1A). For all of the non-NMC cells tested, there was a basal level of BRD4 phosphorylation observed in unsynchronized cells, but BRD4 is hyperphosphorylated in all of the synchronized mitotic cell samples (Figure 1A). BRD4 is also hyperphosphorylated specifically during mitosis in mouse NIH3T3 cells (Figure 1B), indicating that this is a highly conserved phenotype. It is important to note that, unlike the standard SDS/PAGE gels, Phos-tag gels tend to show smeared bands especially for large proteins like BRD4, but the shifts of hyperphosphorylated BRD4 in every gel presented in this study have been confirmed in multiple experimental repeats.

To rule out the possibility that these anti-microtubule drugs artificially trigger BRD4 hyperphosphorylation, we performed mitotic shake-off to isolate the mitotic cells enriched using double thymidine (DT) synchronization and release. As shown in Figure 1C, BRD4 is hyperphosphorylated in DT-enriched mitotic cells to a similar degree as in nocodazole-arrested mitotic cells. To further confirm that BRD4 is hyperphosphorylated during mitosis, mitotic cells collected by DT synchronization and mitotic shake-off were treated with DMSO or proTAME, an anaphase promoting complex (APC) inhibitor that can block mitotic exit. Phos-tag gel analysis showed that BRD4 remained hyperphosphorylated in proTAME-treated cells, which were trapped in mitosis, but became de-phosphorylated in DMSO-treated cells, which were able to exit mitosis (Figure 1D).

Together, our study demonstrates that BRD4 is hyperphosphorylated specifically during mitosis in all human and mouse cells tested. This finding suggests that the mitotic-specific hyperphosphorylation may represent a conserved cellular mechanism regulating BRD4 function during different stages of the cell cycle. However, only in NMC HCC2429 cells, BRD4 is hyperphosphorylated throughout the cell cycle. As suggested by our previous study (44), CDK9 activation could be stimulating BRD4 hyperphosphorylation in NMC cells during interphase, causing the observed phenotype.

### 2.2. CDK1 Is a Potential Kinase That Mediates BRD4 Hyperphosphorylation during Mitosis

**Figure 2:**
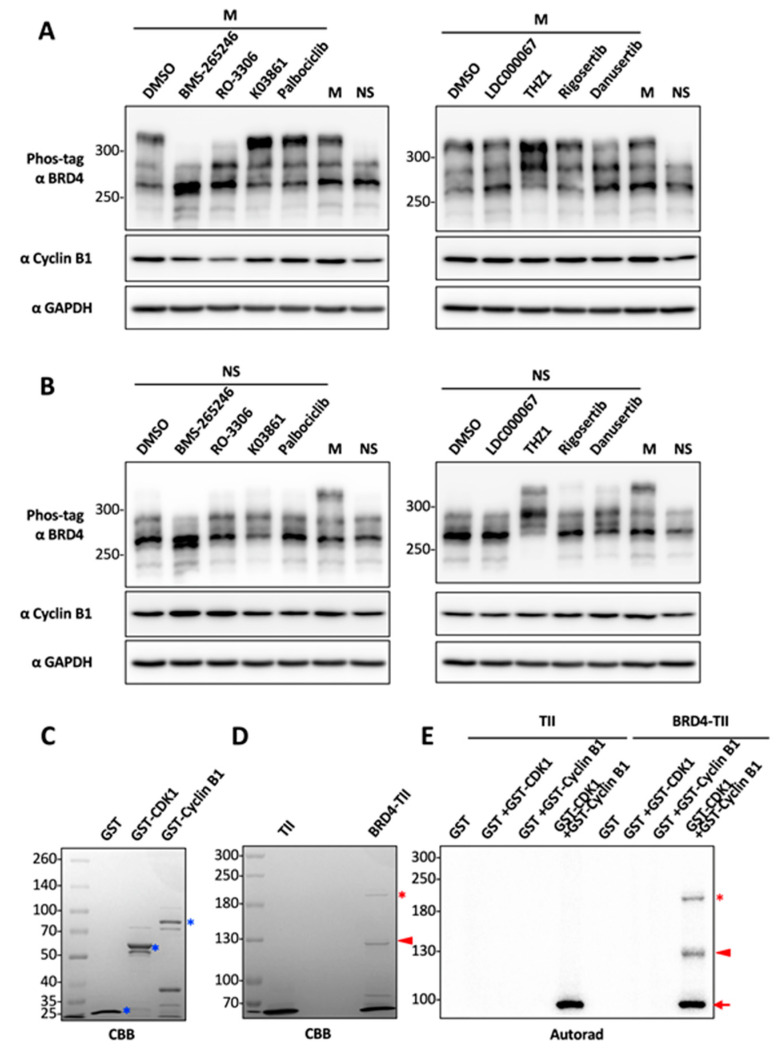
CDK1 is a potential kinase that mediates BRD4 mitotic hyperphosphorylation. (**A**) HEK293T cells were synchronized in mitosis (M) using nocodazole and treated with DMSO or 1 μM of the indicated kinase inhibitors for 1 h. Whole-cell lysates were resolved in Phos-tag gel and immunoblotted with indicated antibody. The untreated mitotic (M) and not-synchronized (NS) cell lysates were loaded on the right as controls. (**B**) HEK293T cells (not synchronized, NS) were treated and analyzed as in (**A**). (**C**) GST, GST-CDK1 and GST-Cyclin B1 proteins purified from *E. coli* were analyzed by SDS-PAGE and Coomassie Brilliant Blue (CBB) staining. Asterisks mark the purified GST or GST fusions. (**D**) TII control and BRD4-TII expressed in *E. coli* were affinity purified using IgG beads, resolved in 5.5% SDS/PAGE and visualized using CBB staining. TII, which has a molecular weight of approximately 16 kDa, has run off the gel. This lane serves as a negative control to identify the BRD4-specific bands in the BRD4-TII lane. Asterisks mark the full length BRD4-TII. Triangles mark a shorter fragment of BRD4-TII. (**E**) Recombinant TII control and BRD4-TII purified with IgG beads were subjected to in vitro kinase assay using purified GST, GST-CDK1, and/or GST-Cyclin B1 as indicated. The samples were analyzed by SDS-PAGE and autoradiography. Asterisks mark the full length BRD4-TII. Triangles mark a shorter fragment of BRD4-TII. The arrow marks phosphorylated GST-Cyclin B1.

To identify the kinase(s) responsible for BRD4 mitotic hyperphosphorylation, we treated HEK293 cells that have been arrested in mitosis with inhibitors of CDK1, 2, 4, 6, 7, and 9, Polo-like kinases (PLKs) 1/2, and Aurora kinase A/B/C. To avoid any cytotoxicity, the cells were only treated with each drug for one hour. Among the inhibitors tested, only RO-3306 (targeting CDK1) and BMS-265246 (mainly targeting CDK1 and 2) abolished BRD4 mitotic hyperphosphorylation (Figure 2A).

BRD4 mitotic hyperphosphorylation was not affected after treatment with K03861 (targeting CDK2), palbociclib (targeting CDK4 and 6), LDC000067 (targeting CDK9), rigosertib (targeting PLK1 and 2), and danusertib (targeting Aurora A/B/C) (Figure 2A). These compounds also did not affect the basal level of BRD4 phosphorylation observed in asynchronous cells. In contrast, the CDK7 inhibitor, THZ1, appeared to promote BRD4 phosphorylation in both unsynchronized and mitotically synchronized cells (Figure 2A,B). Whether CDK7 could regulate a phosphatase activity targeting BRD4 remains to be studied in the future. Together, our data suggest that CDK1 is a potential kinase responsible for the mitotic-specific phosphorylation of BRD4.

In cells treated with BMS-265246 and RO-3306, the Cyclin B1 protein level was slightly reduced. This is consistent with a previous landmark study showing that adding CDK1 inhibitors to cells in mitosis could induce mitotic exit and cyclin B degradation [60]. To avoid this cell cycle effect, we only treated the cells with the indicated kinase inhibitors for 1 h so that RO-3306 and BMS-265246 treatment only caused very minor reduction of Cyclin B1, which could not account for the dramatic inhibition of BRD4 mitotic hyperphosphorylation. However, the data from Figure 2A indicates that this Cyclin B1 reduction could contribute to some degree of loss in BRD4 mitotic hyperphosphorylation. Therefore, to rule out this cell cycle effect, we performed an in vitro kinase assay to test whether CDK1 could directly phosphorylate BRD4. Recombinant GST-CDK1 and GST-Cyclin B1 were expressed and purified from *E. coli* (Figure 2C). In addition, recombinant BRD4 fused to a tobacco etch virus (TEV) protease cleavage site and two IgG binding domains of protein A (TII) [43] was expressed in *E. coli* and immuno-precipitated on IgG beads, whereas the TII protein was similarly purified as a negative control (Figure 2D). BRD4-TII and the TII tag were subjected to the in vitro kinase assay in a reaction mix containing purified GST protein (serving as a negative control), GST-CDK1 and/or GST-Cyclin B1 proteins. Incubation with GST-CDK1, GST-Cyclin B1, or GST alone did not lead to BRD4 phosphorylation in vitro. Only when GST-CDK1 was combined with GST-Cyclin B1, which promotes formation of the active CDK1 kinase complex, was BRD4 phosphorylation clearly detected (Figure 2E). On the other hand, no phosphorylation of the TII protein was detected under any of the conditions tested. Cyclin B1 was clearly phosphorylated by CDK1 in both the BRD4-TII and TII reaction, providing an internal positive control for the CDK1 kinase activity in these reactions. In addition to the kinase assay using the recombinant GST-CDK1 and GST-Cyclin B1 expressed and purified from *E. coli*, we have also performed the BRD4 in vitro phosphorylation assay using recombinant human CDK1/CyclinB1 kinase complex produced by baculovirus-infected Sf9 cells, with which we detected strong and dose-dependent phosphorylation of BRD4 (Figure S2). However, from this assay, we cannot exclude the possibility that other eukaryotic kinases carried over from the insect cells might be present in this kinase sample and contributed to the BRD4 phosphorylation kinase activity. Compared to the CDK1/CyclinB1 kinase complex produced by insect cells, the CDK1/CyclinB1 complex purified from *E. coli* shows relatively weak activity toward BRD4, but it provides clear evidence that the CDK1/Cyclin B1 complex but not other contaminating eukaryotic kinase(s) could directly phosphorylate BRD4 in vitro. These studies therefore identified CDK1 as the potential kinase that phosphorylates BRD4 during mitosis.

### 2.3. Determination of BRD4 Mitotic Phosphorylation Sites by Mutagenesis

**Figure 3:**
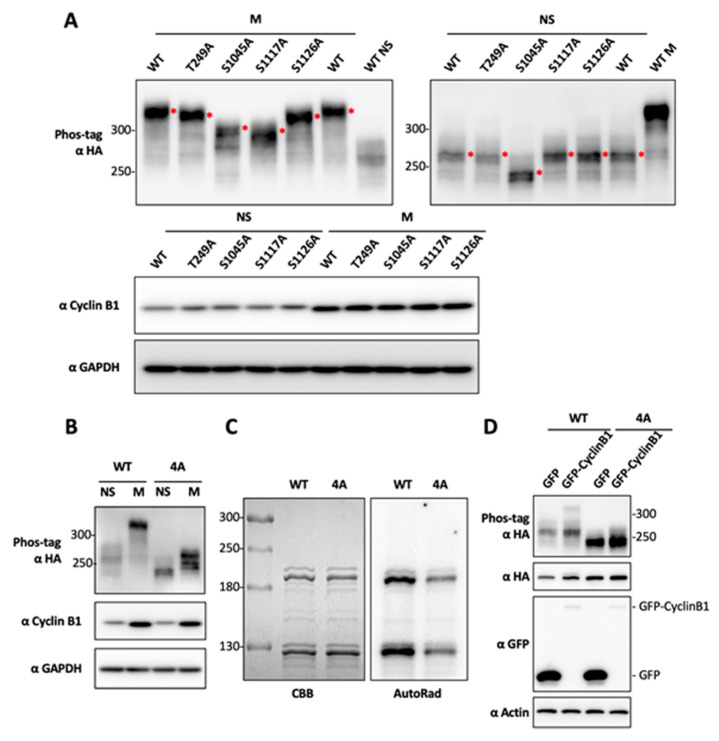
Determination of BRD4 mitotic phosphorylation sites by alanine-substitution mutagenesis. (**A**) HEK293T cells were transfected with plasmids encoding HA-tagged WT BRD4 or single-A substitution mutants. Cells were not synchronized (NS) or synchronized in mitosis (M) using nocodazole. Whole-cell lysates were collected and analyzed on Phos-tag or SDS/PAGE gels and immunoblotted using the indicated antibodies. Asterisks mark the major BRD4 bands in the Phos-tag gel. (**B**) HEK293T cells transfected with plasmids encoding HA-tagged BRD4 WT or 4A mutant were not synchronized (NS) or synchronized in mitosis (M) using nocodazole. Whole-cell lysates were analyzed in Phos-tag or SDS/PAGE gels and immunoblotted using the indicated antibodies. (**C**) BRD4-TII WT or 4A mutant expressed in *E. coli* were affinity purified using IgG beads and subjected to in vitro kinase assay using purified GST-CDK1 and GST-Cyclin B1. The samples were resolved in SDS/PAGE and visualized by autoradiography. (**D**) HEK293T cells were co-transfected with plasmids encoding HA-tagged BRD4 WT or 4A mutant and either the GFP vector control or GFP-Cyclin B1 construct. Whole-cell lysates were analyzed in Phos-tag or SDS/PAGE gels and immunoblotted using the indicated antibodies.

In order to understand the functional impact of BRD4 mitotic hyperphosphorylation, we set out to identify the BRD4 amino acid residues phosphorylated by CDK1 during mitosis. Using the ScanSite online kinase-specific phosphorylation site analysis server [61], we found 21 predicted CDK1 phosphorylation sites on the BRD4 protein. To identify the BRD4 residues that are specifically hyperphosphorylated during mitosis, we performed alanine (A) substitution mutagenesis on these potential phosphorylation sites to determine their impact on BRD4 hyperphosphorylation. We first mutated the thirteen residues recognized by CDK1 on the C-terminal half of BRD4, including T847, S858, S891, T897, T942, S1045, S1070, S1083, S1117, S1126, T1186, T1212 and T1309, to A. To examine the impact of the single-A substitution mutations on BRD4′s phosphorylation status during mitosis, these mutants were individually expressed in HEK293T cells. After harvesting the lysate from mitotic cells, the BRD4 single-A mutants were resolved in Phos-tag gels and analyzed by western blotting. From these studies, the BRD4 mutants S1045A, S1117A and S1126A each showed significantly reduced BRD4 mitotic phosphorylation, as indicated by the faster migrating BRD4 bands compared to wild type (WT) BRD4 protein (Figure 3A, left panel). 

Using a similar approach to analyze the eight predicted CDK1 recognition sites located on the N-terminal half of BRD4, including T204, T210, T221, T236, T249, T278, T296 and S470, we discovered that only the BRD4 T249A mutation moderately inhibits BRD4 mitotic hyperphosphorylation (Figure 3A, left panel). In addition, we analyzed all of the BRD4 single-A mutants in asynchronous cells to determine if they affect the basal level of BRD4 phosphorylation. While T249A, S1117A and S1126A do not show any major effect, S1045A significantly inhibits the basal level of BRD4 phosphorylation in unsynchronized cells (Figure 3A, right panel).

Based on the single-A mutagenesis results, we generated the BRD4 4A mutant containing the T249A, S1045A, S1117A and S1126A mutations. When expressed in HEK293T cells, the BRD4 4A mutant extracted from mitotic cells migrates to the same position as the WT BRD4 protein isolated from the unsynchronized cells (Figure 3B). This result suggests that the 4A mutations eliminate nearly all of BRD4′s mitotic phosphorylation. The BRD4 4A mutant collected from unsynchronized cells migrates further down in the Phos-tag gel likely because the S1045A mutation could abolish the basal level of BRD4 phosphorylation in unsynchronized cells (Figure 3A,B).

Since the BRD4 4A mutations could block BRD4 mitotic hyperphosphorylation in HEK293T cells, we also performed an in vitro kinase assay to determine whether these mutations can prevent CDK1-mediated BRD4 phosphorylation. The autoradiography of the in vitro kinase assay samples show that the BRD4 4A mutant has dramatically reduced levels of CDK1/Cyclin B1-mediated BRD4 phosphorylation compared to WT BRD4 (Figure 3C). To confirm this in vitro result in cells, we expressed BRD4 and GFP-Cyclin B1 in HEK293T cells. While overexpression of Cyclin B1 was sufficient to induce hyperphosphorylation of the wild type BRD4 molecule, BRD4 4A quadruple mutagenesis efficiently abolishes Cyclin B1/CDK1-mediated BRD4 hyperphosphorylation (Figure 3D). Together, these studies identified four BRD4 residues, T249, S1045, S1117, and S1126, as the major sites for CDK1-mediated BRD4 mitotic hyperphosphorylation. Because BRD4 4A quadruple mutagenesis abolishes most but not all of BRD4′s hyperphosphorylation in mitotic cells as well as in the in vitro kinase reaction (Figure 3), it is possible that BRD4 residues other than those represented in the 4A mutant could also be phosphorylated by CDK1 (see Discussion).

### 2.4. CDK1-Mediated BRD4 Hyperphosphorylation Contributes to BETi Resistance

**Figure 4:**
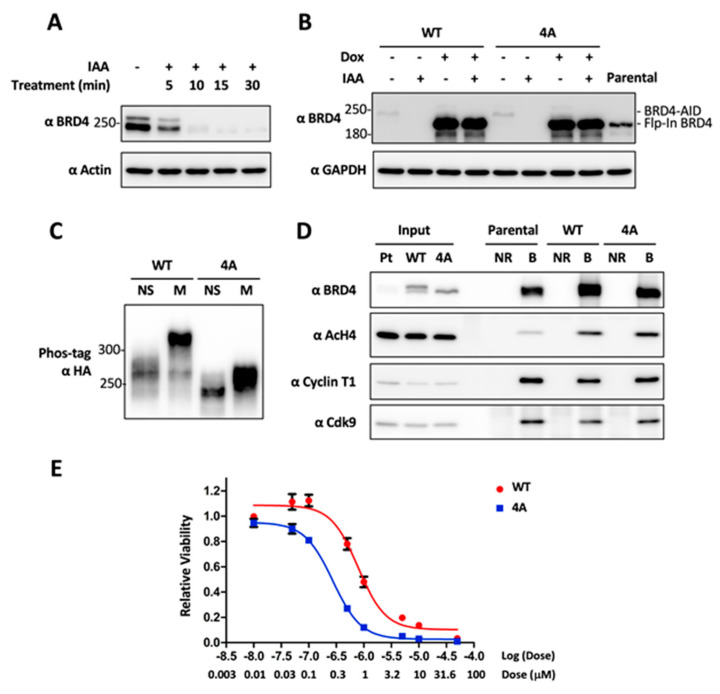
Blocking CDK1-mediated BRD4 hyperphosphorylation confers sensitivity to BETi. (**A**) DLD-1 cells with CRISPRed BRD4-AID were treated with IAA and harvested at the indicated time points. Whole-cell lysates were analyzed in SDS/PAGE gels and immunoblotted using the indicated antibodies. (**B**) DLD-1 parental cells and those with CRISPRed BRD4-AID as well as Flp-In BRD4 WT or 4A were treated with or without Dox and/or IAA as indicated for 1 day. Whole-cell lysates were analyzed in SDS/PAGE gel and immunoblotted using the indicated antibodies. (**C**) DLD-1 cells with CRISPRed BRD4-AID and Flp-In HA-tagged BRD4 WT or 4A were synchronized using the double thymidine method. At the time of the second thymidine release, Dox and nocodazole were added to the cells. After 19 h, whole-cell lysates were analyzed in a Phos-tag gel and immunoblotted with HA antibody. (**D**) DLD-1 parental cells, DLD-1 cells with CRISPRed BRD4-AID and Flp-In BRD4 WT or 4A (treated with Dox and IAA) were synchronized in mitosis with nocodazole treatment after releasing from a single thymidine block. The whole cell lysates were immunoprecipitated with normal rabbit (NR) or BRD4 (B) antibodies. The samples were immunoblotted with the indicated antibodies. (**E**) DLD-1 cells with CRISPRed BRD4-AID and Flp-In BRD4 WT or 4A were incubated with Dox and IAA for 1 day before treating with DMSO or increasing doses of (+)-JQ1. CellTiter-GLO cell viability assays were performed 3 days after (+)-JQ1 treatment. Relative cell viability was determined by normalizing the (+)-JQ1 values to the DMSO control values. The corresponding dose-inhibition curves were analyzed using the nonlinear regression method in Prism software.

Identification of the major BRD4 residues phosphorylated by CDK1 allows us to dissect the impact of CDK1-mediated BRD4 mitotic hyperphosphorylation. In order to specifically examine the function of these four residues without the influences of cell toxicity or the potential off-target effect associated with BRD4 knockdown/knockout by siRNA or CRISPR (clustered regularly interspaced short palindromic repeats), we applied auxin-inducible degron (AID) CRISPR knock-in technology [62,63,64,65] to achieve inducible and transient depletion of BRD4 while expressing exogenous WT or 4A BRD4 using the Flp-In^TM^ T-REx^TM^ reconstitution system (Thermo Fisher Scientific, Waltham, MA, USA). Using DLD-1 cells that show BRD4 mitotic hyperphosphorylation (Figure 1), CRISPR gene editing was first exploited to generate a cell line in which the C-terminus of endogenous BRD4 is fused with AID [63]. This method allows endogenous BRD4 to be degraded within 15 min after auxin treatment (Figure 4A). 

We then used the Flp-In^TM^ T-REx^TM^ reconstitution system to introduce Doxycycline (Dox) inducible expression of WT BRD4 or the non-phosphorylatable 4A mutant in DLD-1/BRD4-AID cells. With this approach, we achieved robust expression of the exogenous BRD4 molecules while endogenous BRD4 is degraded by auxin induction (Figure 4B). Using the established cell lines, we confirmed that the Flp-In WT BRD4 is hyperphosphorylated during mitosis while the 4A mutant is not (Figure 4C). Using these two cell lines, we performed immunofluorescent staining of mitotic chromosomes and mitotic spindles as well as mitotic index analysis to determine how blocking BRD4 mitotic hyperphosphorylation affects mitotic progression. From these studies, we did not detect any obvious difference between WT and 4A BRD4 Flp-In cells regarding mitotic chromosome structure and chromosome segregation dynamics. In addition, immunoprecipitation of WT and 4A BRD4 from DLD-1/BRD4-AID Flp-In mitotic cell lysates showed that BRD4 WT protein and 4A mutant bind to components of the pTEFb complex with similar affinities. These results suggest that the 4A mutation does not significantly affect BRD4′s general ability to recruit transcriptional activators (Figure 4D).

BRD4 phosphorylation has been shown to enhance its binding to acetylated chromatin [43,44,66]. In order to determine the effects of mitotic hyperphosphorylation on BRD4′s affinity for chromatin, we performed a BRD4 co-immunoprecipitation experiment and found that the BRD4 WT protein and 4A mutant could pull down similar amount of acetylated histone H4 (Figure 4D). We also used increasing salt concentrations to extract WT and 4A BRD4 from nuclear lysates as described previously [44]. Through this method, we also did not detect any significant difference in the bulk chromatin binding affinities of WT or 4A BRD4 (Figure S3). Because BRD4 has been shown to function as a mitotic bookmarker preserving the epigenetic memory of key M/G1 growth-associated genes through mitosis to ensure their rapid postmitotic transcriptional re-activation [5,6,7,8], we hypothesized that CDK1-activated BRD4 mitotic hyperphosphorylation allows BRD4 to bind more strongly to its specific bookmarked genes during mitosis to stimulate their expression, promote downstream cellular proliferation, and consequently contribute to BETi resistance [35]. To test this idea, we examined if WT BRD4 Flp-In cells are more resistant to JQ1 inhibition compared to the cells expressing the non-phosphorylatable 4A mutant. Indeed, when the two cell lines were cultured in the presence of increasing doses of the BETi JQ1, the IC50 was reduced from 0.79 uM for WT BRD4 cells to 0.27 uM for the 4A mutant expressing cells (Figure 4E).

### 2.5. Combinatory Inhibition of CDK1 and BRD4 Chromatin Binding Induces a Synergistic Antitumor Effect

**Figure 5:**
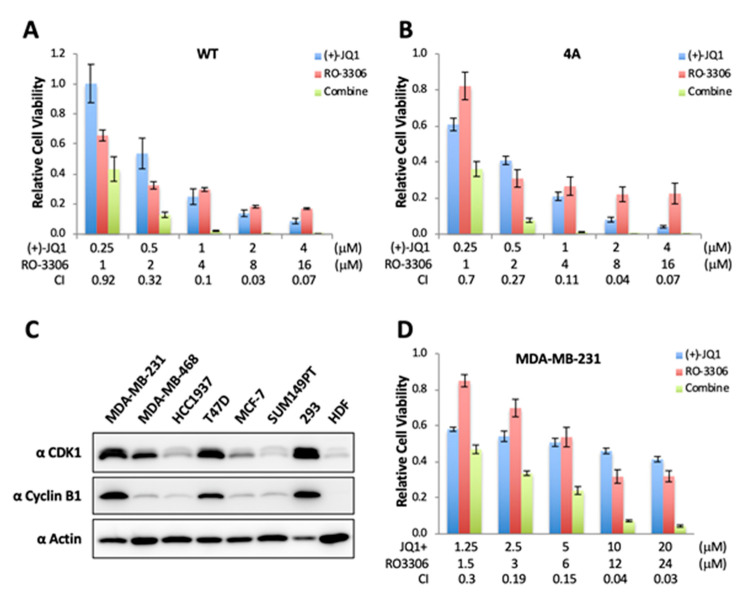
BETi and CDK1 inhibitor show synergistic effects in killing cancer cells. (**A**,**B**) DLD-1 cells with CRISPRed BRD4-AID and Flp-In BRD4 WT (**A**) or 4A (**B**) were incubated with Dox and IAA for 1 day before treating with DMSO or the indicated doses of (+)-JQ1, RO-3306 or a combination of both drugs. CellTiter-GLO cell viability assays were performed 3 days after drug treatment. Relative cell viability was determined by normalizing the drug treatment values to the DMSO control values. (**C**) Whole cell lysates of indicated breast cancer cells together with 293 and HDF cells were resolved in SDS/PAGE and immunoblotted with the indicated antibodies. (**D**) MDA-MB-231 cells were treated with DMSO or the indicated doses of (+)-JQ1, RO-3306 or a combination of both drugs. CellTiter-GLO cell viability assays were performed 3 days after drug treatment. Relative cell viability was determined by normalizing the drug treatment values to the DMSO control values. CI values were analyzed with CompuSyn software.

Although blocking BRD4 interaction with acetylated histones using BETis has been pursued in cancer clinical trials, many cancers have been found to acquire resistance to BETis [34,35,36,37]. However, the underlying mechanism remains poorly understood. We and others found that hyperphosphorylated BRD4 binds much tighter to chromatin [44,66]. Therefore, it is not only more difficult to be dissociated from its target genes by BETi, but also contributes to stronger target oncogene expression that could fortify BETi resistance [35] (Figure 4E). Given our observations that CDK1 inhibition prevents BRD4 hyperphosphorylation and that CDK1-mediated mitotic hyperphosphorylation of BRD4 contributes to BETi resistance (Figure 4E), we reasoned that CDK1 inhibitors used in combination with BETis may be able to generate a synergistic effect in suppressing cancer cell proliferation. To test this hypothesis, we treated DLD-1/BRD4-AID cells expressing Flp-In WT BRD4 or the non-phosphorylated mutant with varying doses of (+)-JQ1 and RO-3306 either individually or in combination, and analyzed the potential synergy of BETis with the CDK1 inhibitor in repressing cancer cell proliferation. Using a previously published method [67], we calculated the combination index (CI) to quantify the synergism of the drug combination. In this method, a given drug combination is considered to be synergistic when the calculated CI value is less than 1, with smaller CI values indicating a stronger synergy of the combination [67]. Individual treatment of (+)-JQ1 and RO-3306 inhibited the growth of BRD4 WT cells in a dose-dependent manner (Figure 5A). 

A synergistic effect was observed with the dual treatment at all of the drug concentrations tested while a greater synergistic effect on the viability of treated cells was observed as the drug doses increased (Figure 5A). Similar synergy was observed in the BRD4 4A cells.

Triple negative breast cancers (TNBCs) are an example of an aggressive cancer with BETi resistance. The TNBC’s BETi resistance is attributed to hyperphosphorylation of BRD4 due to decreased activity of Protein Phosphatase 2A (PP2A) in these cells [35]. Because CDK1 is frequently overexpressed or amplified in cancer [68,69,70,71] and expression of the BRD4 target gene MYC is significantly elevated in related cancers such as TNBCs [72], we hypothesize that CDK1 activated in cancers may stimulate BRD4 hyperphosphorylation to support stronger chromatin binding and target oncogene expression, thereby driving tumor growth and BETi resistance. By analyzing the TCGA transcriptomic dataset, we discovered that CDK1 is highly expressed in the vast majority of TNBC tumors. We therefore decided to test the synergistic effects of CDK1 inhibition with BETi treatment in TNBC cells. We first compared the CDK1 and Cyclin B1 protein levels in several common TNBC cell lines (MDA-MB-231, MDA-MB-468, HCC1937, and SUM149PT) and non-TNBC breast cancer cell lines (T47D and MCF-7) with those in 293 cells and normal HDFs. Compared to the noncancerous HDFs, higher levels of cyclin B1 and/or CDK1 were detected in a number of the cancer cell lines (Figure 5C). The TNBC cell line MDA-MB-231 showed the highest expression of both proteins and was chosen for subsequent experiments (Figure 5C). To measure the synergy of BETis and CDK1 inhibitors in killing these cancer cells, MDA-MB-231 cells were treated with varying concentrations of (+)-JQ1 and the CDK1 inhibitor RO-3306 individually or in combination, and cell viability was measured. While treatment with RO-3306 alone caused a dose-dependent inhibition of MDA-MB-231 cell growth, these cells appeared to be more resistant to (+)-JQ1 compared to DLD-1 cells discussed above (Figure 5D). However, combined treatment with RO-3306 and (+)-JQ1 achieved a strong synergistic effect (CI value of <0.3) on MDA-MB-231 viability even at the lowest dosage tested (Figure 5D). These results suggest that inhibition of BRD4 hyperphosphorylation with CDK1 inhibitors could be a viable strategy for overcoming cancer cell resistance to BETis.

## 3. Discussion

Previous studies from our group and others have revealed important functions of BRD4 in cellular growth control [1,2,3,4,9,10,12,13,38,39,40,41,73]. Dysregulation of BRD4 contributes to the pathogenesis of a wide range of cancers [4,14,15,16,17,18,19,20,21,22,23,24,25,26,27]. BRD4 has thus emerged as a key anti-cancer therapeutic target. However, very little is known about how BRD4 functional activity is regulated in normal cells and how dysregulation of BRD4 function contributes to tumorigenesis. Hence, it has been challenging to develop anti-cancer strategies that specifically target BRD4 oncogenic activity without affecting its normal function in noncancerous cells. In this study, we discovered that BRD4 is hyperphosphorylated during mitosis and identified CDK1 as a potential kinase mediating BRD4 mitotic hyperphosphorylation. Thus, our study reveals a novel mechanism that could potentially regulate BRD4 biological function.

During a compound screen to identify small molecules that could affect BRD4′s phosphorylation status, we identified seven anti-microtubule agents that could efficiently induce BRD4 hyperphosphorylation in HEK293 cells. Our further analysis showed that BRD4 is hyperphosphorylated specifically during mitosis (Figure 1). By analyzing a panel of inhibitors targeting mitotic kinases, we found that only CDK1 inhibitors can specifically block BRD4 mitotic hyperphosphorylation (Figure 2), suggesting that CDK1 could be responsible for BRD4 mitotic-specific hyperphosphorylation.

BMS-265246 can inhibit CDK1/Cyclin B, CDK2/Cyclin E, and CDK4/Cyclin D with IC50 of 6 nM, 9 nM, and 0.23 μM, respectively [74]. On the other hand, RO-3306 selectively inhibits CDK1/cyclin B1 activity with a Ki value of 35 nM [75]. Therefore, BMS-265246 inhibits CDK1/Cyclin B1 with an IC50 that is nearly 10 times lower than RO-3306. This much stronger effectiveness of BMS-265246 in inhibiting CDK1/Cyclin B1 may contribute to its stronger effect in blocking BRD4 mitotic hyperphosphorylation as shown in Figure 2A. The effect of BMS-265246 observed in Figure 2 also suggests that other targets of BMS-265246, such as CDK2 and CDK4, could be involved in BRD4 mitotic hyperphosphorylation. To rule out this possibility, we have tested K03861 (selectively inhibits CDK2 with Kd of 50 nM) and palbociclib (selectively inhibits CDK4/6 with IC50 of 11 nM/16 nM) in multiple experimental repeats, which consistently showed that K03861 and palbociclib could not inhibit BRD4 mitotic hyperphosphorylation (Figure 2A and Figure S4). Our finding therefore suggests that CDK2 and CDK4 are unlikely to contribute to BRD4 mitotic hyperphosphorylation. BMS-265246 belongs to the Type I family of kinase inhibitors, which are ATP-competitors that mimic the purine ring of the adenine moiety in ATP. The targeted ATP pocket is highly conserved throughout the kinome, making Type I inhibitors more likely to show low kinase selectivity with high potential for off-target effects [76]. As described above, the higher potency of BMS-265246 in competing the kinase ATP-binding sites as compared to RO-3306 is likely to reduce its specificity, thereby targeting other unknown kinase(s), which could contribute to BRD4 phosphorylation. In addition, dual treatment of RO-3306 (CDK1 inhibitor) and K03861 (CDK2 inhibitor) could not achieve the same effect in blocking BRD4 phosphorylation as the treatment with BMS-265246, which inhibits both CDK1 and CDK2 (Figure S5), further supporting that BMS-265246 may target other kinases, which could phosphorylate BRD4. In addition, CDK1 inhibition could also cause abnormal mitotic exit [60], thereby indirectly reducing BRD4 mitotic hyperphosphorylation. To rule out this possibility, we performed the in vitro BRD4 phosphorylation assay using CDK1 and Cyclin B1 purified from *E. coli* (Figure 2). In this reaction, the recombinant CDK1/Cyclin B1 complex was able to phosphorylate BRD4, providing direct evidence to support that CDK1 is the potential kinase phosphorylating BRD4 during mitosis.

To fully understand the function of CDK1-mediated BRD4 mitotic hyperphosphorylation, we mutated all 21 predicted CDK1 phosphorylation sites on the BRD4 protein. By analyzing the BRD4 mutants in both mitotic cells as well as in the in vitro kinase assay, we identified four BRD4 residues, T249, S1045, S1117, and S1126, to be critical for CDK1-mediated BRD4 mitotic hyper- phosphorylation. By combining auxin-inducible degron and Flp-In^TM^ T-REx^TM^ reconstitution technologies, we were able to generate stable cell lines in which endogenous BRD4 is replaced by Dox-induced WT BRD4 or the non-phosphorylatable 4A mutant. Using these cell lines, we first tested if blocking BRD4 mitotic hyperphosphorylation induces defective mitosis. We did not observe any obvious defect of mitotic chromosome segregation, such as unaligned/lagging/bridging chromosomes or multipolar spindles, in the BRD4 4A cells. This result suggests that BRD4 mitotic hyperphosphorylation may not play a role in mitotic progression. However, we also noticed that BRD4 4A quadruple mutagenesis abolishes most but not all of BRD4′s hyperphosphorylation in mitotic cells as well as in the in vitro kinase reaction (Figure 3), suggesting that other BRD4 residues could also be phosphorylated by CDK1 in mitosis to contribute to BRD4′s mitotic function. Future studies will aim to identify and block the phosphorylation of these additional BRD4 residues to determine if BRD4 mitotic hyperphosphorylation regulates chromosome segregation and mitotic progression.

We discovered that BRD4 is normally hyperphosphorylated only during mitosis and quickly becomes dephosphorylated as the cells exit mitosis (Figure 1). This finding suggests that BRD4 hyperphosphorylation is tightly controlled to support its biological function in normal cells and that dysregulation of BRD4 phosphorylation could contribute to BRD4′s oncogenic activities. We and others have shown that hyperphosphorylated BRD4 binds much tighter to chromatin [44,66], making it more resistant to dissociation from chromatin by BETi, and contributing to stronger target oncogene expression that could fortify BETi resistance. In support of this notion, we showed that blocking the CDK1 phosphorylation sites on BRD4 makes the cells more sensitive to JQ1 treatment (Figure 4E and Figure 5B). In addition, we found that overexpression of CDK1 or its functional partner, Cyclin B1, stimulates BRD4 hyperphosphorylation outside of mitosis in asynchronous cells (Figure 3D). CDK1 is frequently overexpressed or activated in cancer cells [68,69,70,71]. We have also found that BRD4 is hyperphosphorylated throughout the cell cycle in many different types of cancers [43]. In addition, both CDK1 upregulation and BRD4 hyperphosphorylation have been observed in BETi-resistant cancer cells [35]. Based on these findings, we hypothesized that dysregulated CDK1 activation in cancer cells may trigger aberrant BRD4 hyperphosphorylation that persists outside of mitosis, supporting stronger chromatin binding and aberrant induction of its mitotic-bookmarked oncogenes, thereby driving tumor growth and BETi resistance. Building on this line of reasoning, we speculated that combination of a CDK1 inhibitor and BETi can synergistically inhibit cancer cell growth. Indeed, when MDA-MB-231 TNBC cells were treated with (+)-JQ1 and RO-3306, we observed a synergistic effect in repressing cell viability. Similar effect was observed when the dual treatment was tested in DLD-1/BRD4-AID cells expressing either WT or 4A Flp-In BRD4. In addition, we noticed that the cells expressing the non-phosphorylatable BRD4 4A mutant become moderately more sensitive to (+)-JQ1 treatment (Figure 4E and Figure 5B), possibly because blocking CDK1-mediated BRD4 mitotic hyperphosphorylation could reduce its affinity for its mitotic bookmarked genes, making it more easily dissociated by BET inhibitors. On the other hand, these cells also become slightly more resistant to RO-3306 (Figure 5B), likely because the 4A mutagenesis prevents CDK1-mediated phosphorylation on these residues. When combined, these two opposing effects may contribute to the similar synergism observed in DLD-1/BRD4-AID cells expressing WT and 4A mutant BRD4 (Figure 5A,B). The observation that the BRD4 WT and 4A mutant cells were similarly susceptible to RO-3306 and BETi combination treatment also suggests that CDK1 has other functions in BETi-resistant oncogenesis in addition to its role in phosphorylating BRD4. As discussed above, it is also possible that BRD4 residues other than those represented in the 4A mutant could still be hyperphosphorylated by CDK1, since BRD4 4A quadruple mutagenesis did not completely abolish the ability of BRD4 to be phosphorylated by CDK1 (Figure 3).

A marked increase of BRD4 phosphorylation has been observed in TNBC cells resistant to BETi [35]. In line with this finding, we discovered that, compared to the DLD-1 cell lines, MDA-MB-231 TNBC cells were much more resistant to (+)-JQ1 treatment (Figure 5D). Importantly, combination treatment of MDA-MB-231 cells with (+)-JQ1 and RO-3306 resulted in a much stronger synergistic effect on cell viability compared to those observed in DLD-1 cells (Figure 5D). These results suggest that inhibition of BRD4 hyperphosphorylation with CDK1 inhibitors could be a viable strategy for overcoming cancer cell resistance to BETis.

In summary, our study provides new insights for understanding how BRD4′s function is regulated in normal cells by CDK1-mediated mitotic hyperphosphorylation. Based on this finding, it will be interesting to investigate how aberrant CDK1 activation in cancer cells can be explored as a therapeutic target for treating BRD4-associated cancers. Though blocking BRD4′s interaction with acetylated histones using BETis has been pursued as the principal treatment strategy for BRD4-driven cancers, BETi resistance has emerged in the vast majority of cancers [34,35,36,37]. A previous study has shown that inactivation of PP2A in TNBC contributes to BRD4 hyper-phosphorylation and resistance to BETi [35]. Our finding suggests that CDK1 over-activation in cancers could also contribute to BETi resistance by stimulating BRD4 hyperphosphorylation and downstream target gene expression. Both CDK1 inhibitors and BETi have been explored in clinical trials for cancer therapy [32,33,71,77]. However, low selectivity and a lack of mechanism of action have led to the failure of CDK1 inhibitors, whereas BETi resistance emerged in certain cancers has dampened its efficacy. The synergistic effect of the CDK1 inhibitor and BETi observed in our studies therefore provides the rationale for exploring aberrant CDK1 activation in cancer cells as a therapeutic target to overcome tumor BETi resistance and improve treatments for a diverse array of BRD4-associated cancers. The combinatorial inhibition of CDK1 and BRD4 has the potential to overcome the low specificity of CDK1 inhibitor as well as cancer resistance to BETi, allowing more effective killing of BRD4-driven cancer cells.

## 4. Materials and Methods

### 4.1. Recombinant Plasmid Constructs

pOZN-BRD4 WT (encoding HA-FLAG-BRD4), pET23a(+)-TII (encoding a TII tag control), pET23a(+)-BRD4-TII (encoding full length BRD4 with TII tag, which includes one TEV cleavage site and two IgG binding domains, on the C-terminus) have been described in our previous study [43,78]. For pGEX-6p-1-CDK1 (encoding GST-CDK1) and pGEX-6p-1-CCNB1 (encoding GST-Cyclin B1) construction, the CDK9 and CCNB1 ORFs were PCR amplified from the reverse transcription products of HDF mRNA and subcloned into the pGEX-6p-1 vector. BRD4 mutants were generated by bridge PCR using primers introducing the mutated sites and subcloned into the pOZN vector. All plasmid constructs were verified by DNA sequencing.

### 4.2. Cell Culture and Transfection

293, 293T, DLD-1, HeLa and NIH3T3 cells were maintained in DMEM medium (Invitrogen, Waltham, MA, USA) with 10% FBS (HyClone, Marlborough, MA, USA). HCC2429 (a gift from Thao P. Dang, Vanderbilt University, Nashville, TN, USA) cells were maintained in RPMI 1640 medium (Invitrogen) with 10% FBS (HyClone). U2OS cells were maintained in McCoy’s 5A medium (Invitrogen) with 10% FBS (HyClone). HAP1 cells were maintained in IMDM medium (Invitrogen) with 10% FBS (HyClone). HDFs were prepared as described in our previous study [79] and were maintained in DMEM with 10% FBS. 293T cells were transiently transfected using Lipofectamine 2000 reagent (Invitrogen) following the manufacturer’s instructions.

### 4.3. Phos-tag Gel Technique

The Phos-tag gels containing 10 μM Phos-tag acrylamide AAL-107 (Wako Chemicals, Richmond, VA, USA) and 40 μM MnCl_2_ were prepared following the manufacturer’s instructions. Whole cell lysates were prepared as described in our previous study [43]. The proteins from the lysates were resolved in 5% PAGE Phos-tag gels at 10 mA/gel for 5 h. The gels were washed with transfer buffer containing 4 mM EDTA for more than 20 min and then with transfer buffer containing 0.1% SDS for at least 5 min before transferring onto PVDF membranes. The membranes were immunoblotted with the primary antibody as indicated in the figures. For all Phos-tag gels presented in this study, standard High Range Protein Ladder (Cat. No. 26625, Thermo Fisher Scientific, Waltham, MA, USA) were resolved in each gel along with protein samples tested. However, as indicated by the manufacturer, the molecular weight markers are frequently distorted during Phos-tag gel electrophoresis, and therefore they can only be used as rough estimate of the molecular weights.

### 4.4. In Vitro Kinase Assay

The assay was performed based on a published protocol [80] with minor modification in the ratio of hot and cold ATP. BRD4-TII affinity purified on IgG sepharose (GE Healthcare, Marlborough, MA, USA) was incubated in 30 μL kinase buffer containing 50 mM Tris (pH 7.5), 1 mM DTT, 1 mM MnCl_2_, 5 mM MgCl_2_, 1 μM cold ATP, and 10 μCi [γ-32P] ATP (3000 Ci/mmol, Perkin Elmer, Waltham, MA, USA) supplemented with protease inhibitors and phosphatase inhibitors. Purified recombinant GST, GST-CDK1, and/or GST-CCNB1 proteins were also included in the reaction as indicated in the figure legends. The kinase reaction mixtures were incubated at 30 °C for 1 h with shaking. The beads were washed and boiled in 2× SDS/PAGE sample buffer. The eluted proteins from the beads were resolved on SDS/PAGE gels. After drying on Heto Dry GD-1 gel dryer, the gels were exposed to a phosphor screen and detected using a Typhoon FLA 7000 imager (GE Healthcare).

### 4.5. Chemical Inhibitors

BMS-265246 (SelleckChem, Houston, TX, USA), RO-3306 (SelleckChem), K03861 (SelleckChem), LDC000067 (SelleckChem), THZ1 (SelleckChem), Rigosertib (ApexBio, Houston, TX, USA) and Danusertib (ApexBio) powders were dissolved in DMSO to a stock concentration of 10 mM. The chemical stock solutions were stored as small aliquots at -80 °C.

### 4.6. Western Blot Analyses

The primary antibodies used in the western blot analyses include anti-BRD4N (targeting BRD4 156–284, 1:40,000), anti-BRD4C (targeting BRD4 1313–1362, 1:40,000), anti-Cyclin B1 (1:500, sc-245, Santa Cruz Biotechnology, Dallas, TX, USA), anti-GAPDH (1:4000, G8140-01, US Biological, Salem, MA, USA), anti-Actin (MAB1501, Millipore, Burlington, MA, USA) and anti-HA-HRP (1:2000, 12013819001, Roche, Rotkreuz, Switzerland). HRP-linked anti-rabbit IgG (1:3000; 7074S; Cell Signaling Technology, Danvers, MA, USA) and HRP-linked anti-mouse IgG (1:3000; 7076S; Cell Signaling Technology) were used as secondary antibodies. Western blots were developed using SuperSignal West Pico Chemiluminescent Substrate (Thermo Fisher Scientific, Waltham, MA, USA), and images were captured using an Amersham Imager 600 (GE Healthcare). Detailed information about western blot can be found in Figures S6 and S7.

### 4.7. Generation of DLD-1 Cells with CRISPRed BRD4-AID and Flp-In BRD4 WT or 4A

To construct the plasmids used in the generation of the DLD-1 CRISPRed BRD4-AID cells, the AID-P2A-NeoR repair template was constructed using NEBuilder HIFI DNA Assembly Master Mix (E2621, NEB, Ipswich, MA, USA). Briefly, pUC19 was digested with EcoRI and HindIII. The 5′ homologous arm and 3′ homologous arm regions (more than 800 bp each) were PCR amplified from DLD-1 TIR1 genomic DNA. AID was PCR amplified from the pUC19-EGFP-AID-CENP-A construct [81]. P2A-NeoR was PCR amplified from the pUC19-CENP-A-SNAP-P2A-NeoR construct [81]. The 5′ homologous arm, AID, P2A-NeoR, 3′ homologous arm, and pUC19 fragments were assembled with the NEBuilder HIFI DNA Assembly Master Mix. The sgRNA/Cas9n (D10A) plasmids targeting BRD4 ORF C-terminal were constructed by annealing oligos and then ligating them into pX335 [82] at the BbsI sites. The following oligo pairs were designed: g9 forward 5′- CAC CGA TAT TGA CAA TAG ATC ACT C- 3′ and g9 reverse 5′- AAA CGA GTG ATC TAT TGT CAA TAT C -3′ targeting 23bp upstream the stop codon of the BRD4 gene; g5 forward 5′- CAC CGC TTT TCT GAG CGC ACC TAG G -3′ and g5 reverse 5′- AAA CCC TAG GTG CGC TCA GAA AAG C -3′ targeting 11bp downstream the stop codon of the BRD4 gene. All plasmids were verified by sequencing. To generate the DLD-1 CRISPRed BRD4-AID cell line, the linearized AID-P2A-NeoR repair template DNA, pX335 g9 and pX335 g5 plasmid DNAs were co-transfected into DLD-1 TIR1 parental cells [83] using Lipofectamine 2000 (Invitrogen). Five days after transfection, 0.8 mg/mL G418 was added and cells were cultured under G418 selection for 11 days. Colonies that survived G418 selection were picked and cultured in 96-well plates for expansion and identification. Monoclonal cells with the homozygous genotype of both BRD4 alleles fused to AID-P2A-NeoR were verified by PCR and genomic DNA sequencing.

To construct the plasmids used for the generation of DLD-1 Flp-In BRD4 WT or 4A cells, HA-FLAG-BRD4 WT or 4A DNA fragments generated using PCR amplification from pOZN-BRD4 WT or 4A templates were inserted into pcDNA5/FRT/TO vector (Thermo Fisher) using Hind III and Not I sites. All plasmids were verified by sequencing. To generate the DLD-1 CRISPRed BRD4-AID and Flp-In BRD4 WT/4A cell lines, the pcDNA5/FRT/TO BRD4 WT or 4A plasmid and pOG44 (Thermo Fisher) plasmid were co-transfected into DLD-1 CRISPRed BRD4-AID cells. Four days after transfection, 153 μg/mL hygromycin was added and the cells were cultured under hygromycin selection for 17 days. Surviving cells with Flp-In BRD4 WT or 4A were expanded and verified by Dox induction, western blotting and immunofluorescent staining.

### 4.8. Viability Assay

Flp-In BRD4 WT or 4A cells were treated with 0.5 μM indole-3-acetic acid (IAA) and 50 ng/mL Dox for 1 day before incubation with DMSO control or different concentration of (+)-JQ1. After 3 days, cell viability assays were performed using CellTiter-GLO 2.0 (Promega, Madison, WI, USA) according to the manufacturer’s instruction. Relative viability was calculated by normalization to the DMSO control. The corresponding dose-inhibition curves were analyzed using nonlinear regression method, log(inhibitor) vs. response-Variable slope function in the GraphPad Prism 5.0 software (San Diego, CA, USA).

For the drug combination assays, MDA-MB-231 cells were treated with DMSO or indicated doses of (+)-JQ1 and/or RO-3306, individually or in combination; Flp-In BRD4 WT or 4A cells were treated with 0.5 μM IAA and 50 ng/mL Dox for 1 day before incubation with DMSO control or indicated doses of (+)-JQ1 and/or RO-3306 individually or in combination. 3 days after drug treatment, cell viability assays were performed using CellTiter-GLO 2.0 (Promega) according to the manufacturer’s instruction. Relative cell viability was determined by normalizing the drug treatment values to the DMSO control values. CI values were analyzed with CompuSyn software [67].

## 5. Conclusions

Our work demonstrates that BRD4 is hyperphosphorylated specifically during mitosis in normal cells and identifies CDK1 as the key responsible kinase. Four major residues of BRD4, including T249, S1045, S1117, and S1126, were identified to be phosphorylated by CDK1, contributing to BRD4′s hyperphosphorylation during mitosis. Furthermore, our results suggest that CDK1-mediated BRD4 hyperphosphorylation contributes to BETi resistance, and that inhibition of CDK1 could synergistically work with BETi to more effectively kill cancer cells. Our study thus identifies CDK1 inhibition as a novel strategy to overcome BETi resistance in cancer. Further studies will be aimed at identifying additional key phosphorylation sites on BRD4 that contribute to its hyperphosphorylation during mitosis and oncogenesis, and at dissecting the role of CDK1-mediated BRD4 hyperphosphorylation in oncogenesis.

## Supplementary Materials

The following are available online at https://www.mdpi.com/2072-6694/12/6/1637/s1, Figure S1: Treatment with anti-microtubule agents induces BRD4 hyperphosphorylation, Figure S2: BRD4 could be phosphorylated with insect-expressed CDK1/Cyclin B1 in vitro, Figure S3: BRD4 WT and 4A show similar affinity to chromatin, Figure S4: CDK1 is a potential kinase that mediates BRD4 mitotic hyperphosphorylation, Figure S5: BMS-265246 treatment shows more effect on NUT midline carcinoma-specific BRD4 hyperphosphorylation than combined treatment of RO-3306 and K03861, Figure S6: Uncropped immunoblots and gel images, Figure S7: Densitometry reading data.
