# Supplementary material for: Bromodomain-Containing Protein BRD4 Is Hyperphosphorylated in Mitosis
Source: Cancers (Basel). 2020 Jun 20;12(6):1637. doi: 10.3390/cancers12061637 (PMC7353023; doi:10.3390/cancers12061637)
Supplement: Supplementary file 1 [file cancers-12-01637-s001.pdf]

cancers

Supplementary Files

Bromodomain-Containing Protein BRD4 is Hyperphosphorylated in Mitosis

Ranran Wang, June F. Yang, Flora Ho, Erle S. Robertson and Jianxin You

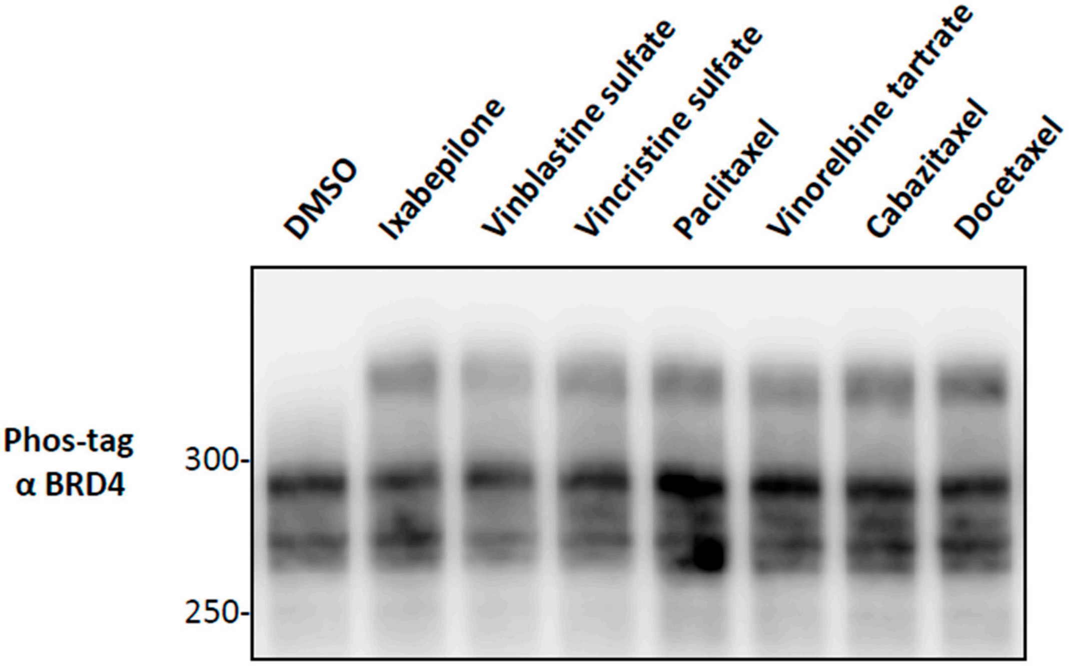

Figure S1. Treatment with anti-microtubule agents induces BRD4 hyperphosphorylation. HEK293 cells were treated with DMSO or 10 μM Ixabepilone, Vinblastine sulfate, Vincristine sulfate, Paclitaxel, Vinorelbine tartrate, Cabazitaxel or Docetaxel for 4 h. Cells were boiled in SDS sample buffer, analyzed in a Phos-tag gel and immunoblotted with BRD4 antibodies.

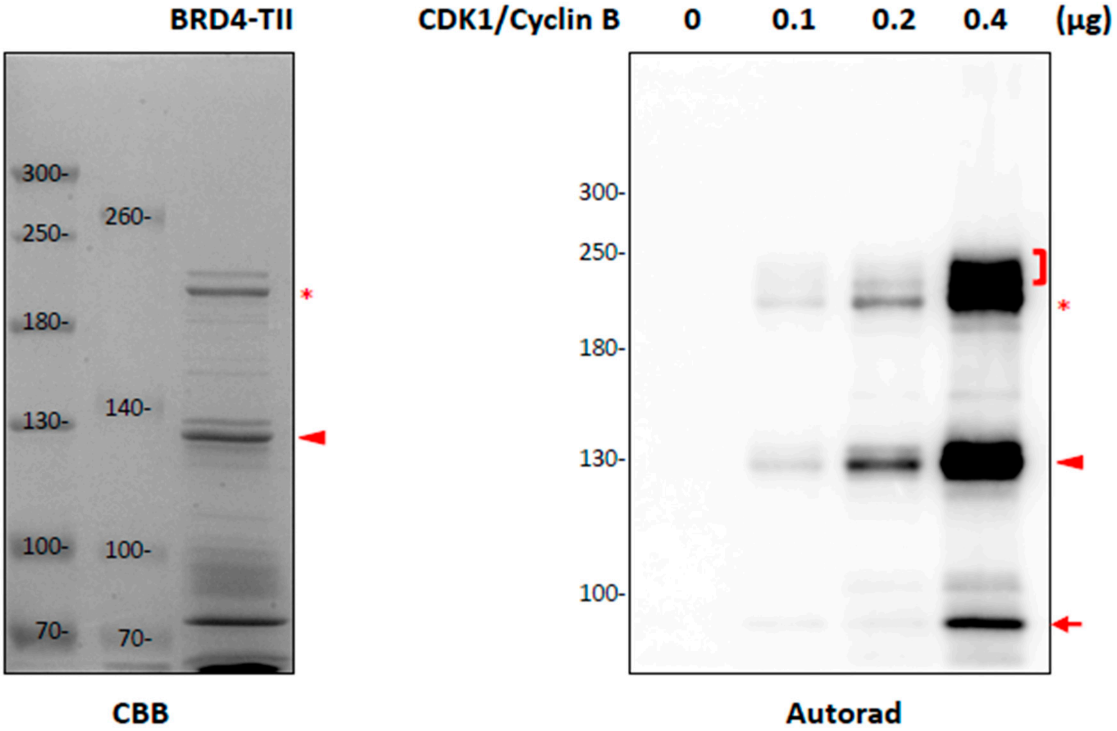

Figure S2. BRD4 could be phosphorylated with insect expressed CDK1/Cyclin B1 in vitro. BRD4-TII expressed in E. coli were affinity purified using IgG beads, resolved in 5.5% SDS/PAGE and visualized using CBB staining (Left panel). BRD4-TII purified with IgG beads were subjected to in vitro kinase assay using insect expressed activated CDK1/Cyclin B1 (Cat. No. 14–450, Millipore). The samples were analyzed by SDS-PAGE and autoradiography (right panel). Asterisks mark the full length BRD4-TII. The bracket marks the hyperphosphorylated BRD4 bands. Triangles mark a shorter fragment of BRD4-TII. The arrow marks phosphorylated Cyclin B1.

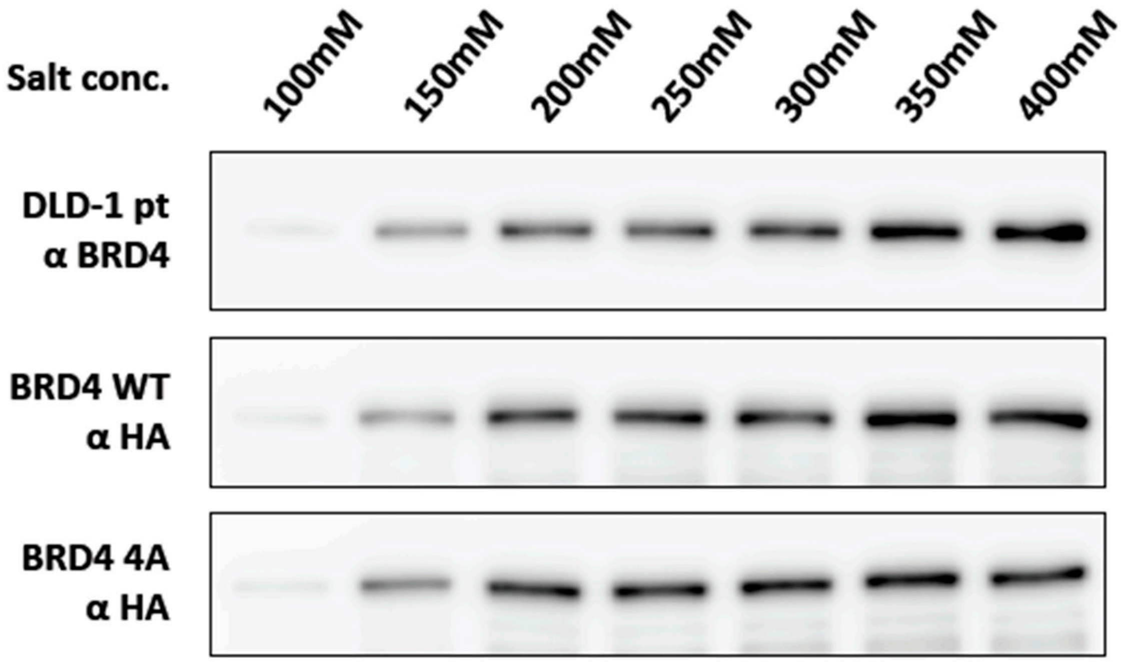

Figure S3. BRD4 WT and 4A show similar affinity to chromatin. Nuclear proteins from DLD-1 parental cells, BRD4 WT or 4A cells (treated with Dox and IAA for 1 d) were extracted with different NaCl concentrations (100–400 mM) and immunoblotted with BRD4 or HA antibodies.

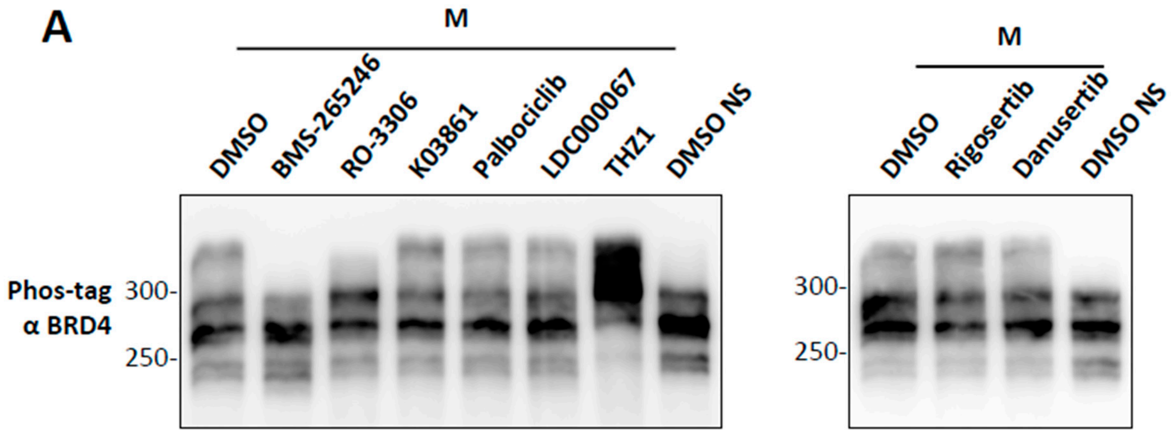

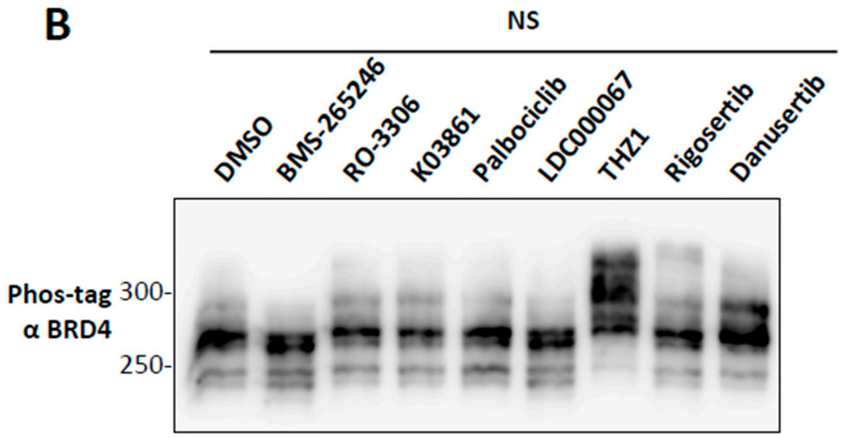

Figure S4. CDK1 is a potential kinase that mediates BRD4 mitotic hyperphosphorylation. (**A**). HEK293 cells were synchronized in mitosis (M) with nocodazole and treated with DMSO or 1 μM of the indicated kinase inhibitors for 1 h. Whole-cell lysates were resolved in Phos-tag gel and immunoblotted with BRD4 antibody. (**B**). HEK293 cells (not synchronized, NS) were treated and analyzed as in (**A**).

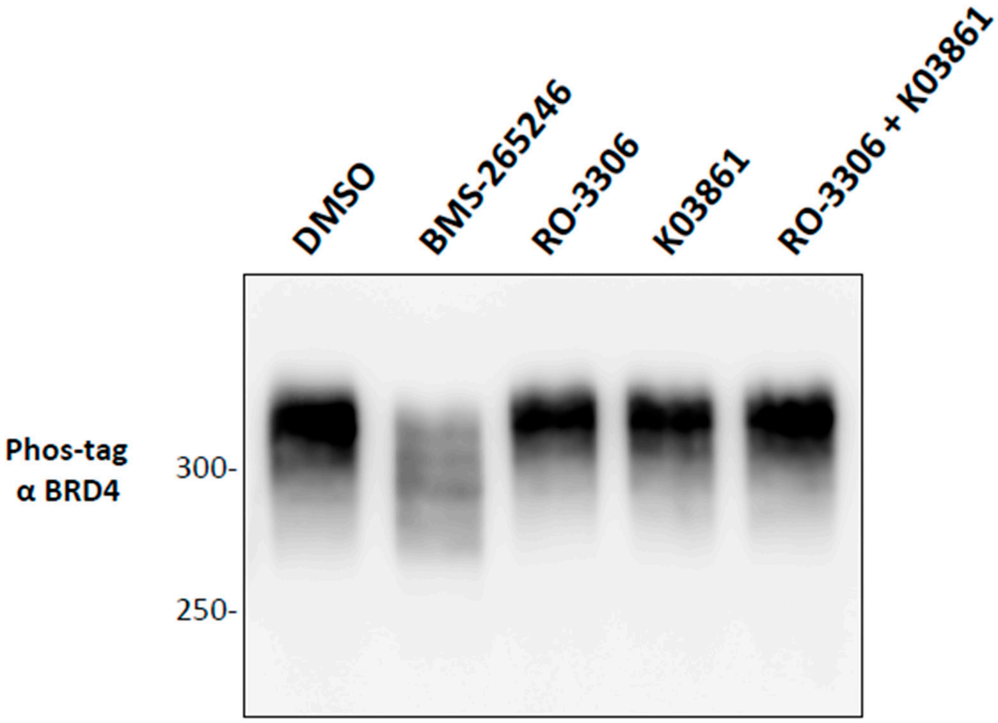

Figure S5. BMS-265246 treatment shows more effect on NUT midline carcinoma-specific BRD4 hyperphosphorylation than combined treatment of RO-3306 and K03861. HCC2429 cells were treated with DMSO or 2 μM of the indicated kinase inhibitors for 1 h. Whole-cell lysates were resolved in Phos-tag gel and immunoblotted with BRD4 antibody.

Figure 1A

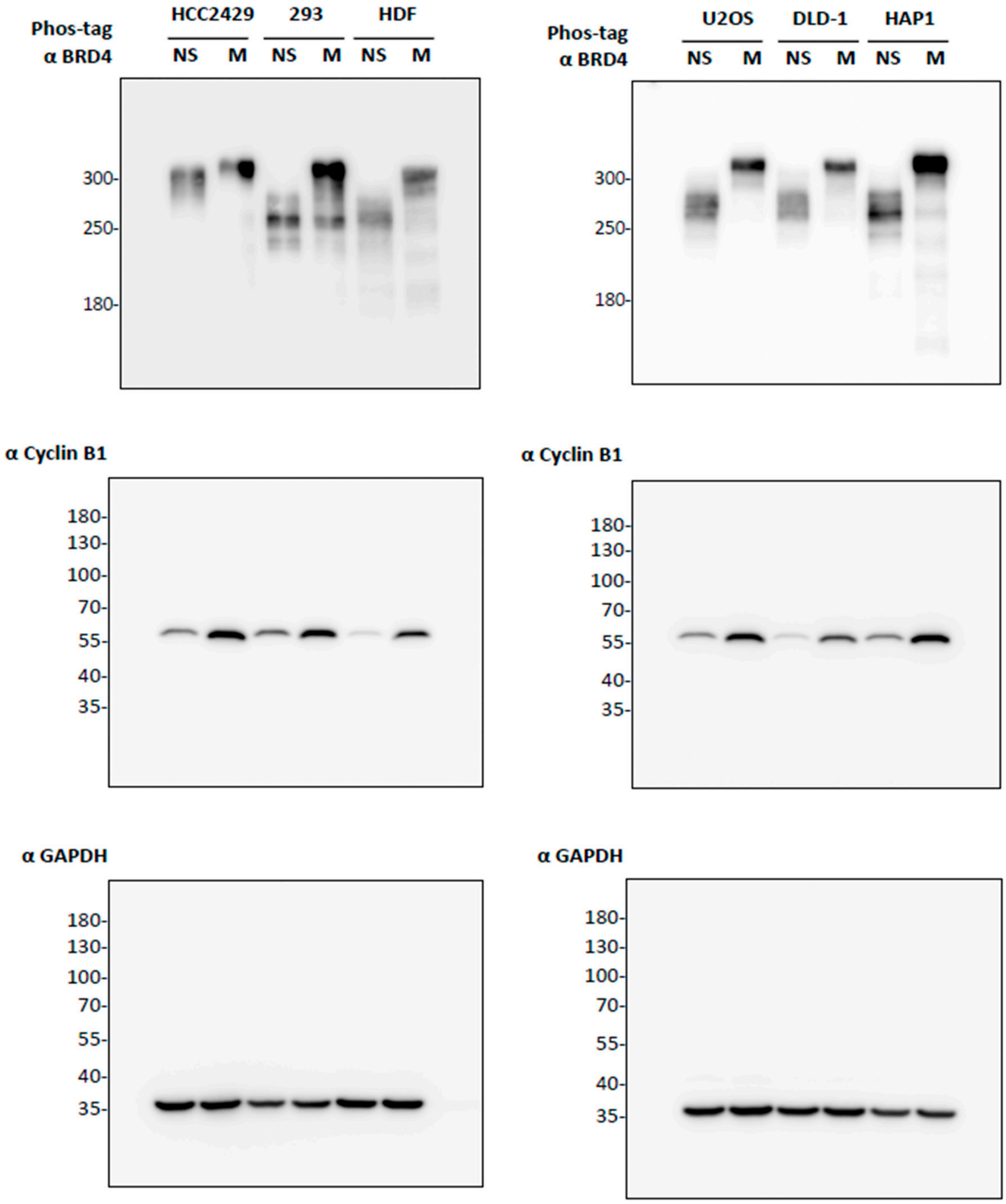
Phos-tag
α BRD4
HCC2429
293
HDF
NS
M
NS
M
NS
M
300-
250-
180-
Phos-tag
α BRD4
U2OS
DLD-1
HAP1
NS
M
NS
M
NS
M
300-
250-
180-
α Cyclin B1
180-
130-
100-
70-
55-
40-
35-
α Cyclin B1
180-
130-
100-
70-
55-
40-
35-
α GAPDH
180-
130-
100-
70-
55-
40-
35-
α GAPDH
180-
130-
100-
70-
55-
40-
35-

Figure 1B

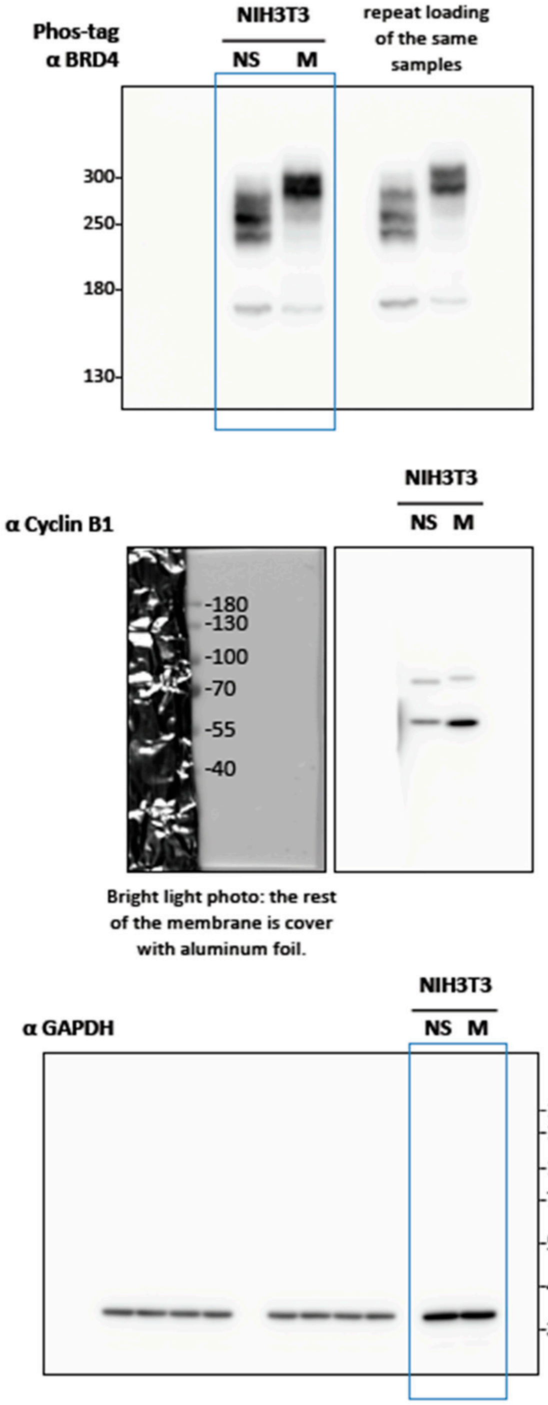

Figure 1C

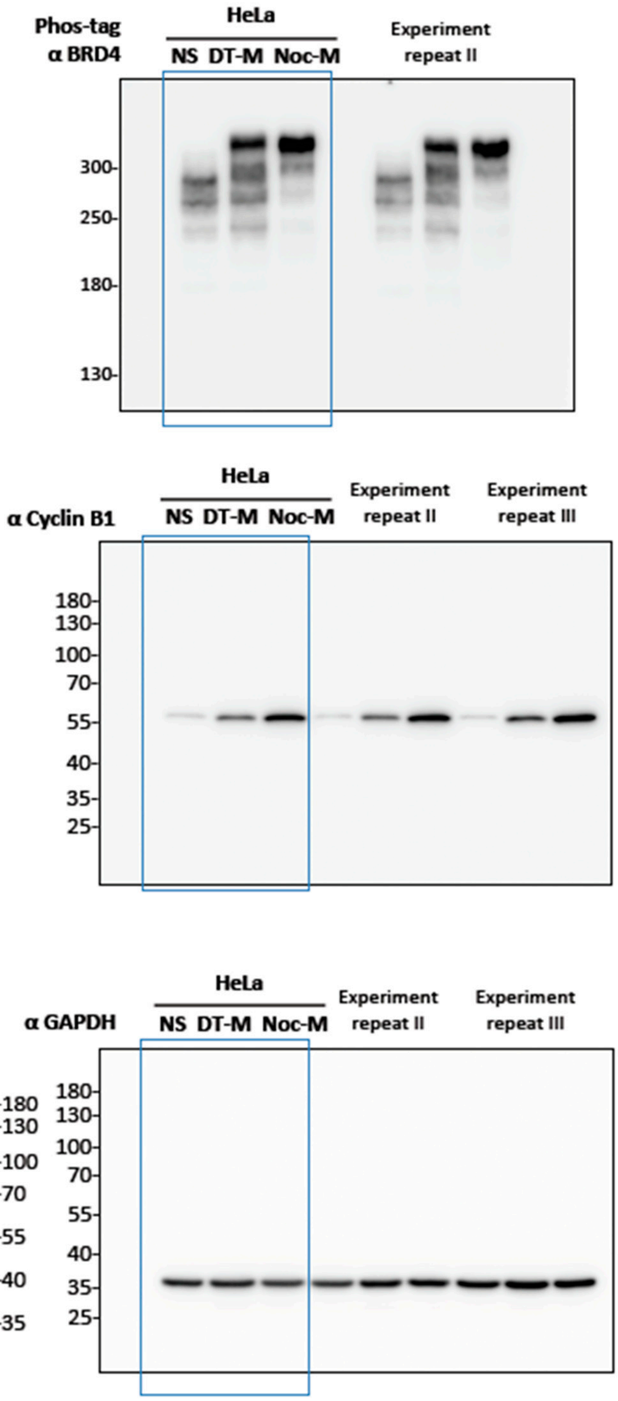

Figure 1D

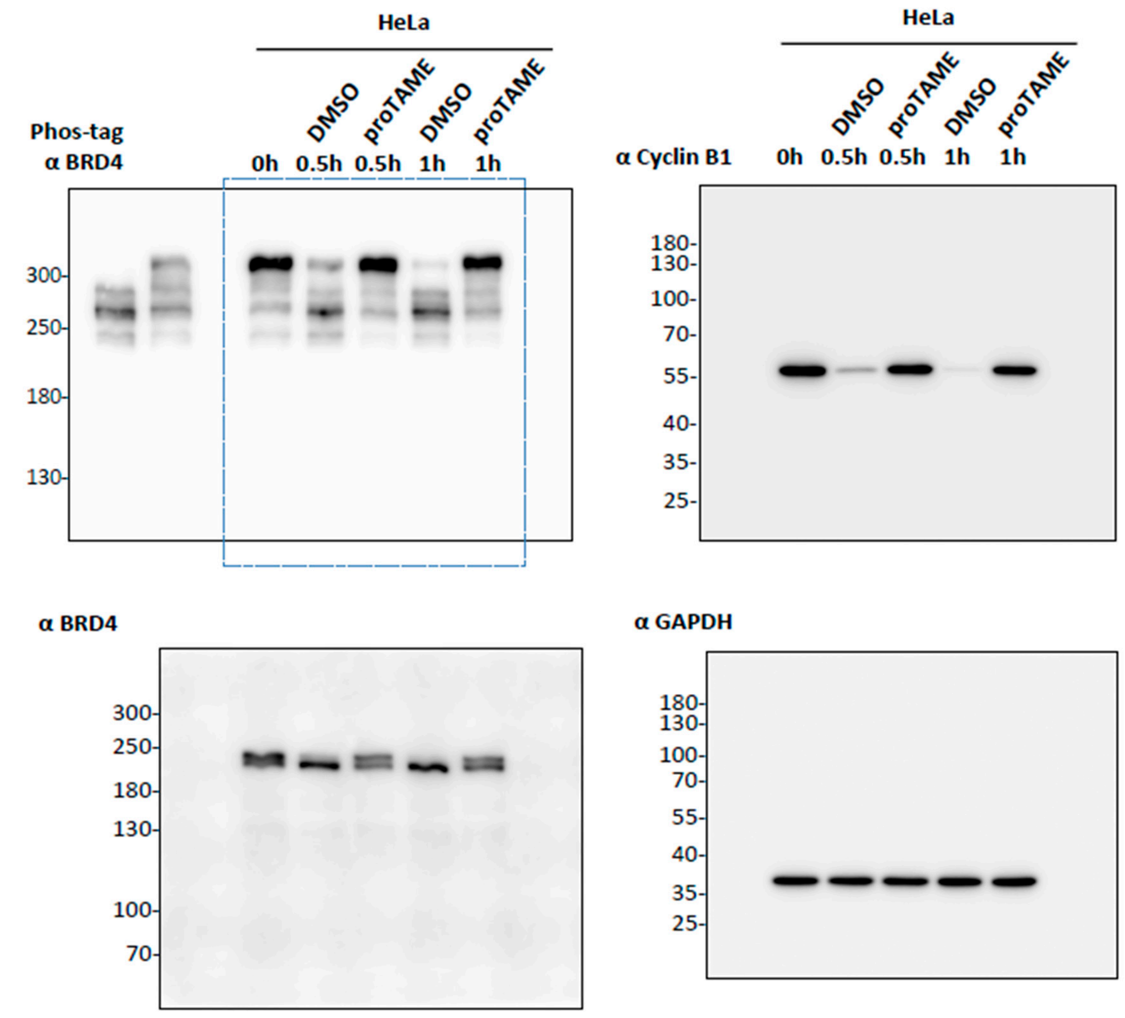
HeLa
Phos-tag
α BRD4
DMSO
proTAME
DMSO
proTAME
0h
0.5h
0.5h
1h
1h
300-
250-
180-
130-
HeLa
α Cyclin B1
DMSO
proTAME
DMSO
proTAME
0h
0.5h
0.5h
1h
1h
180-
130-
100-
70-
55-
40-
35-
25-
α BRD4
300-
250-
180-
130-
100-
70-
α GAPDH
180-
130-
100-
70-
55-
40-
35-
25-

Figure 2A

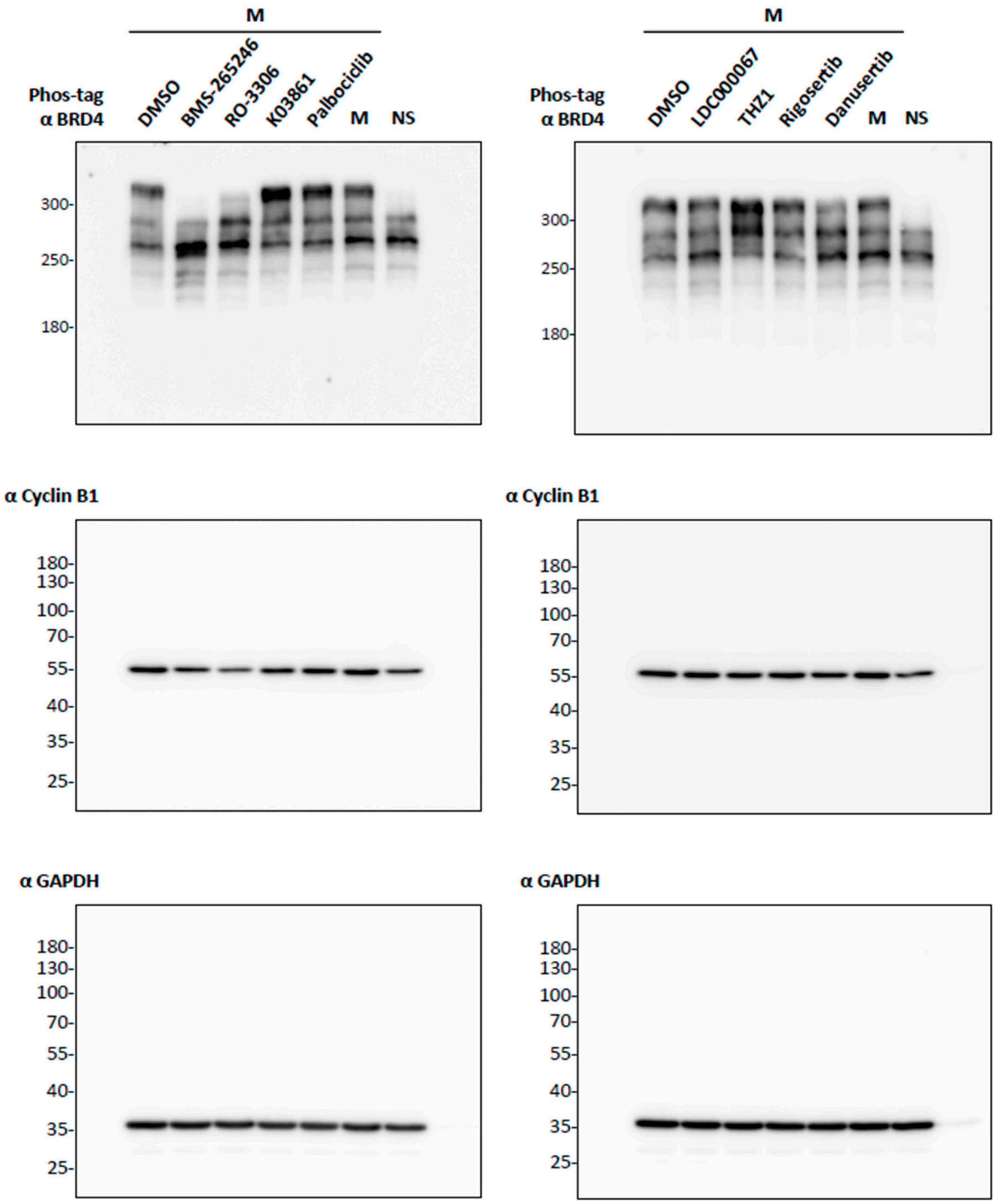
M
Phos-tag
α BRD4
DMSO
BMS-265246
RO-3306
K03861
Palbociclib
M
NS
300-
250-
180-
M
Phos-tag
α BRD4
DMSO
LDC000067
THZ1
Rigosertib
Danusertib
M
NS
300-
250-
180-
α Cyclin B1
180-
130-
100-
70-
55-
40-
35-
25-
α Cyclin B1
180-
130-
100-
70-
55-
40-
35-
25-
α GAPDH
180-
130-
100-
70-
55-
40-
35-
25-
α GAPDH
180-
130-
100-
70-
55-
40-
35-
25-

Figure 2B

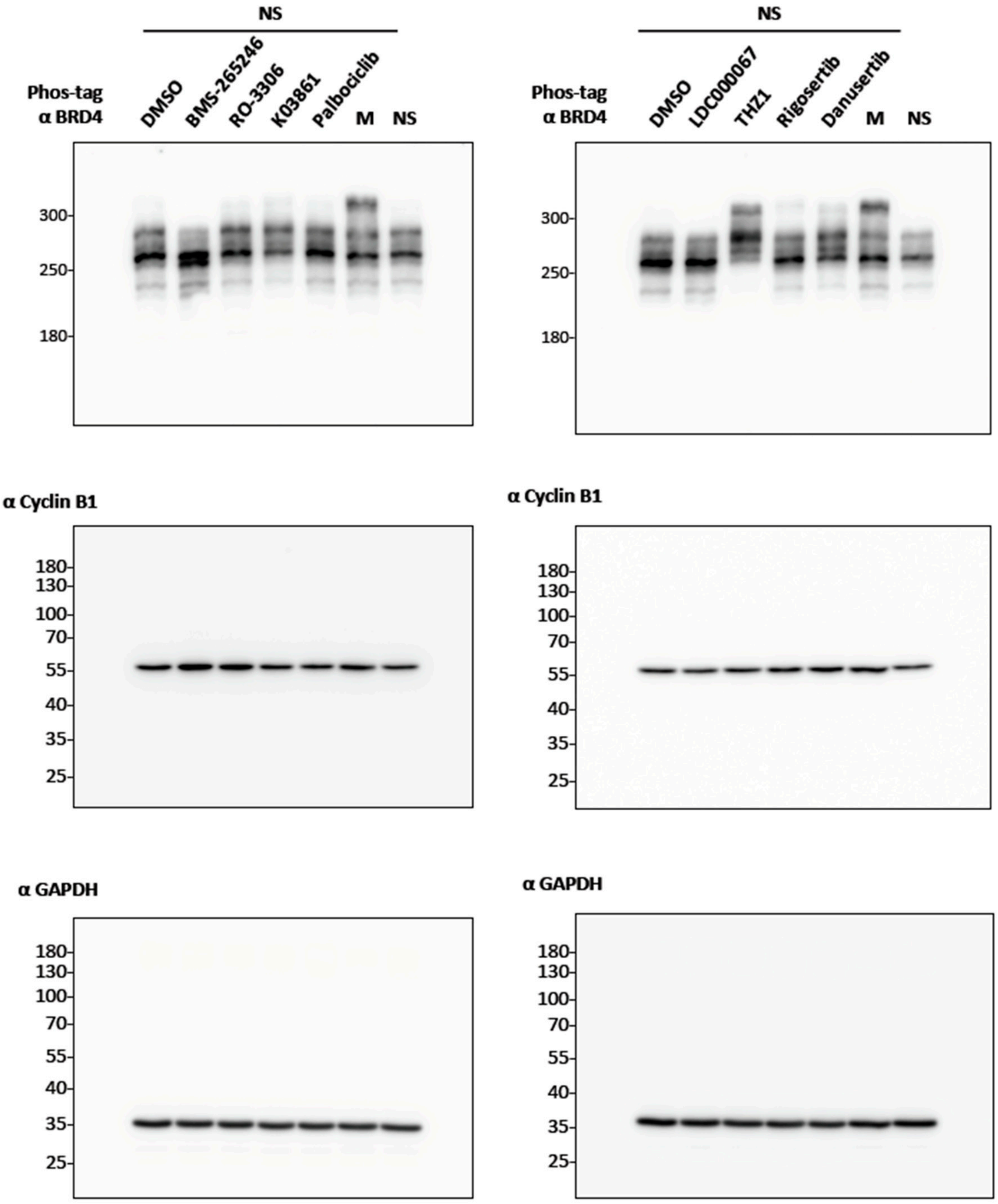
NS
Phos-tag
α BRD4
DMSO
BMS-265246
RO-3306
K03861
Palbociclib
M
NS
300
250
180
NS
Phos-tag
α BRD4
DMSO
LDC000067
THZ1
Rigosertib
Danusertib
M
NS
300
250
180
α Cyclin B1
180
130
100
70
55
40
35
25
α Cyclin B1
180
130
100
70
55
40
35
25
α GAPDH
180
130
100
70
55
40
35
25
α GAPDH
180
130
100
70
55
40
35
25

Figure 2C

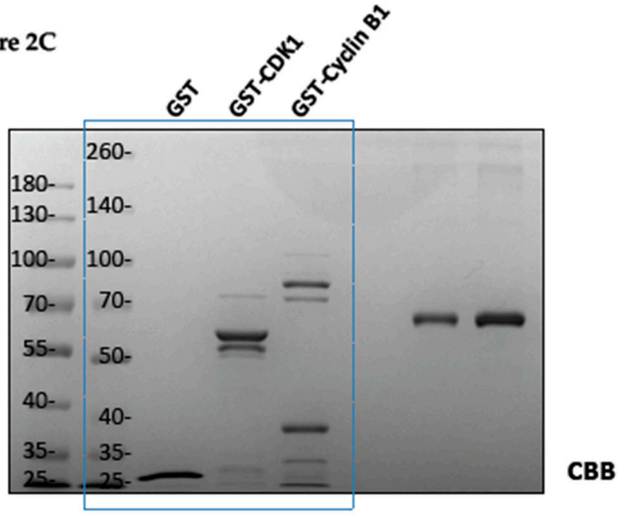

Figure 2D

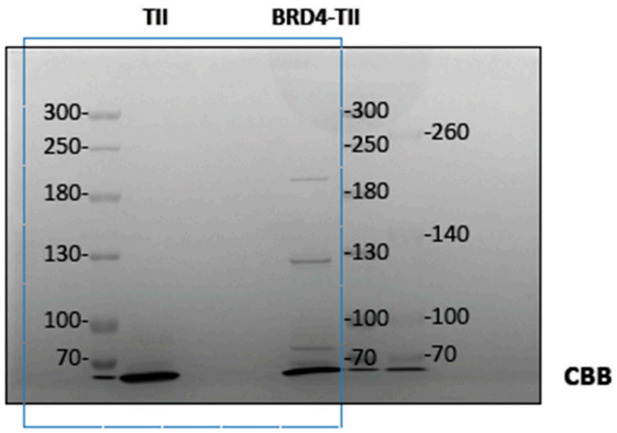

Figure 2E

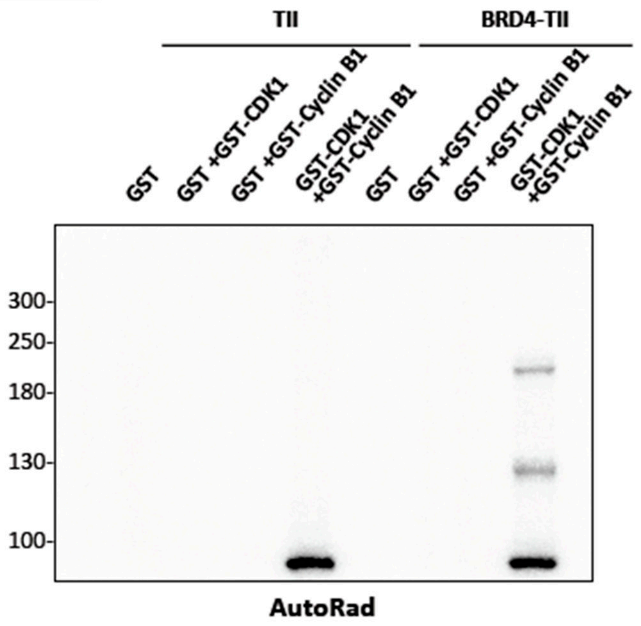

Figure 3A

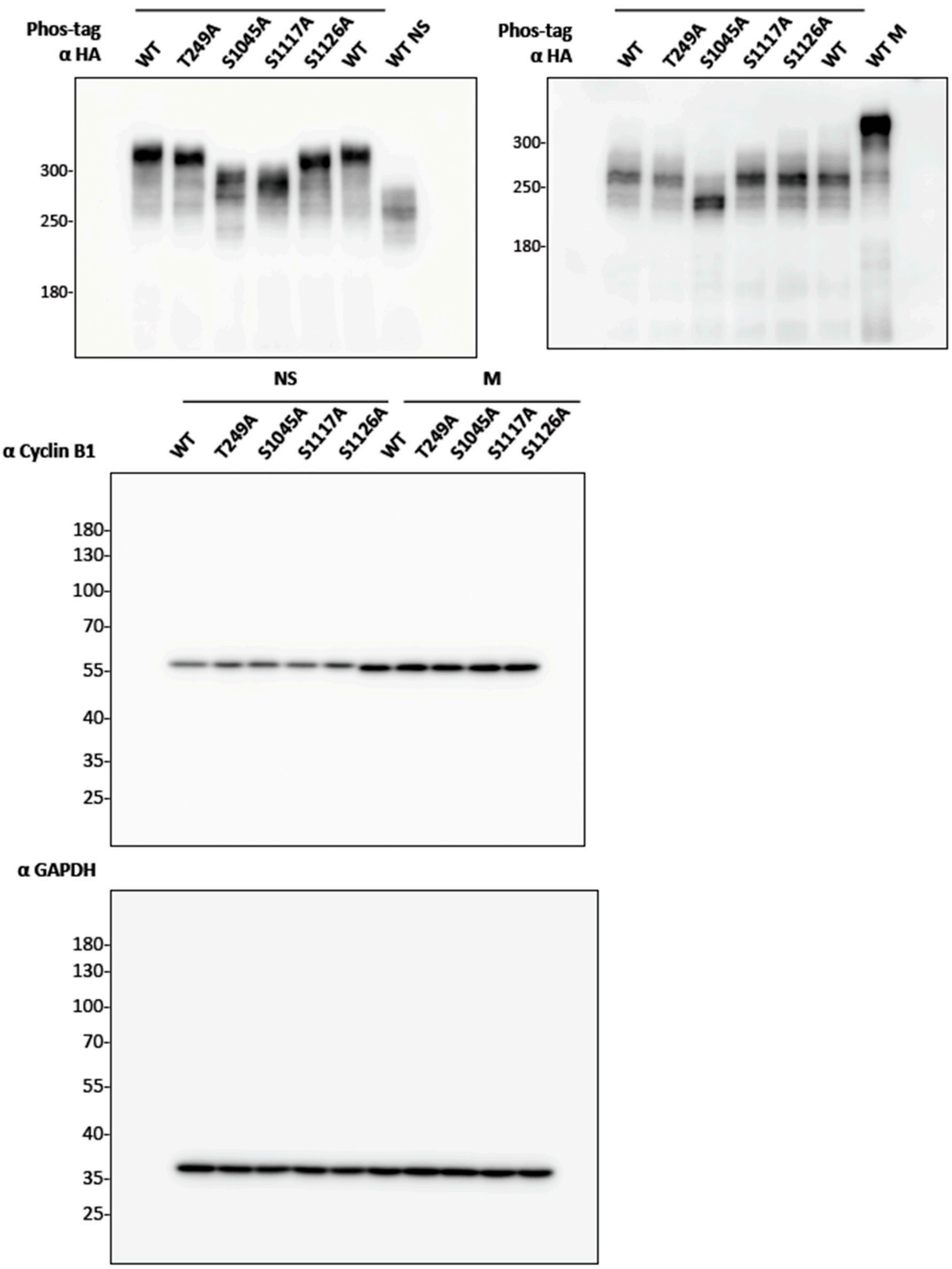
M
Phos-tag
α HA
WT
T249A
S1045A
S1117A
S1126A
WT
WT NS
300-
250-
180-
NS
Phos-tag
α HA
WT
T249A
S1045A
S1117A
S1126A
WT
WT M
300-
250-
180-
NS
M
α Cyclin B1
WT
T249A
S1045A
S1117A
S1126A
WT
T249A
S1045A
S1117A
S1126A
180-
130-
100-
70-
55-
40-
35-
25-
α GAPDH
180-
130-
100-
70-
55-
40-
35-
25-

Figure 3B

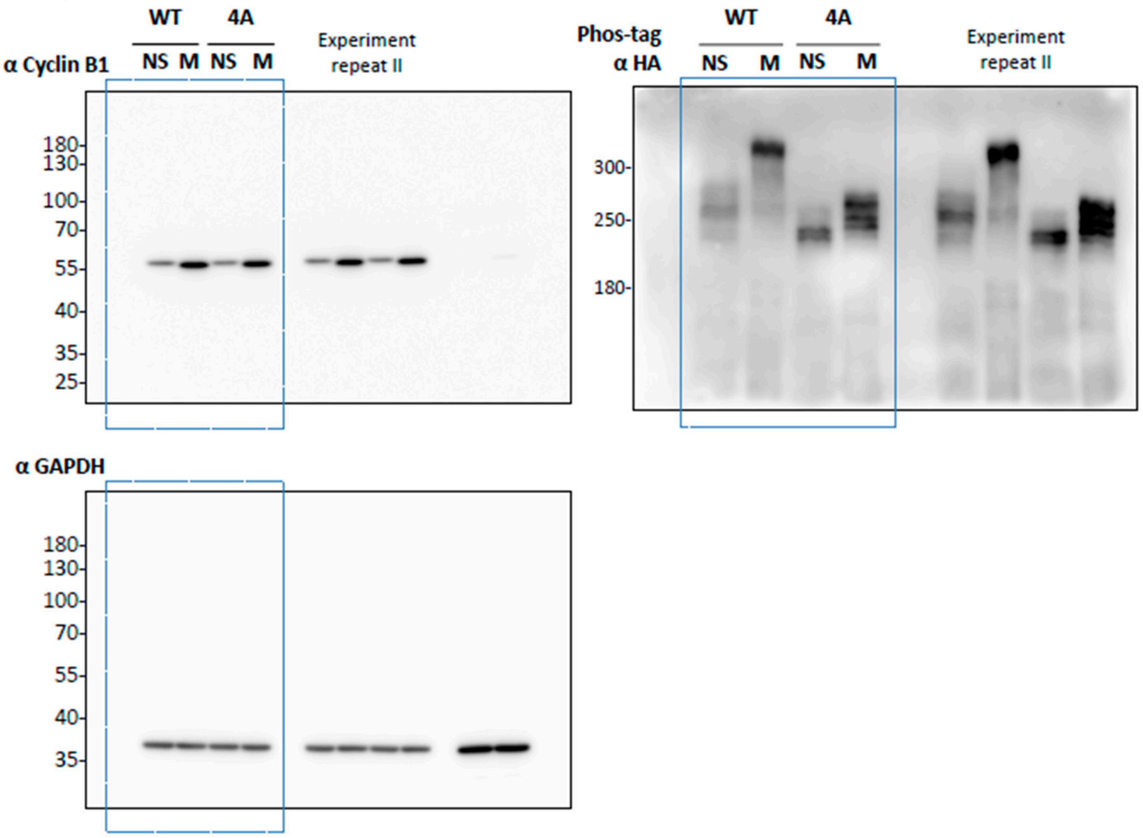

Figure 3C

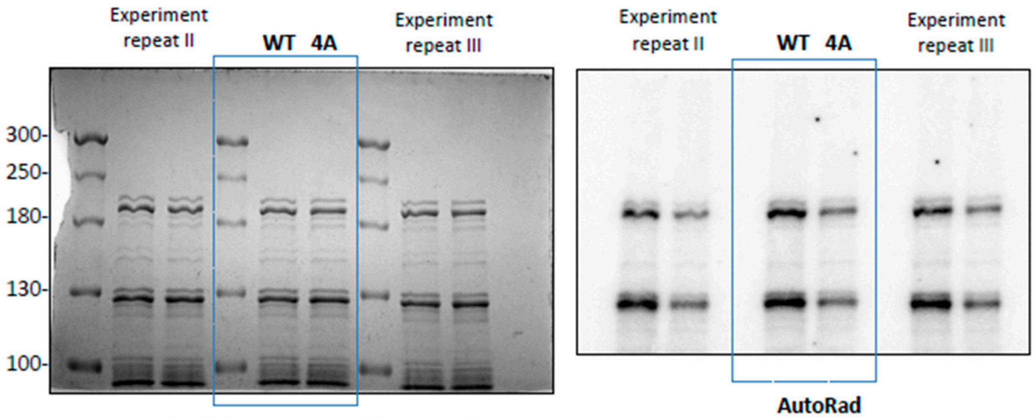

CBB stain of the same gel with AutoRad

AutoRad

Figure 3D

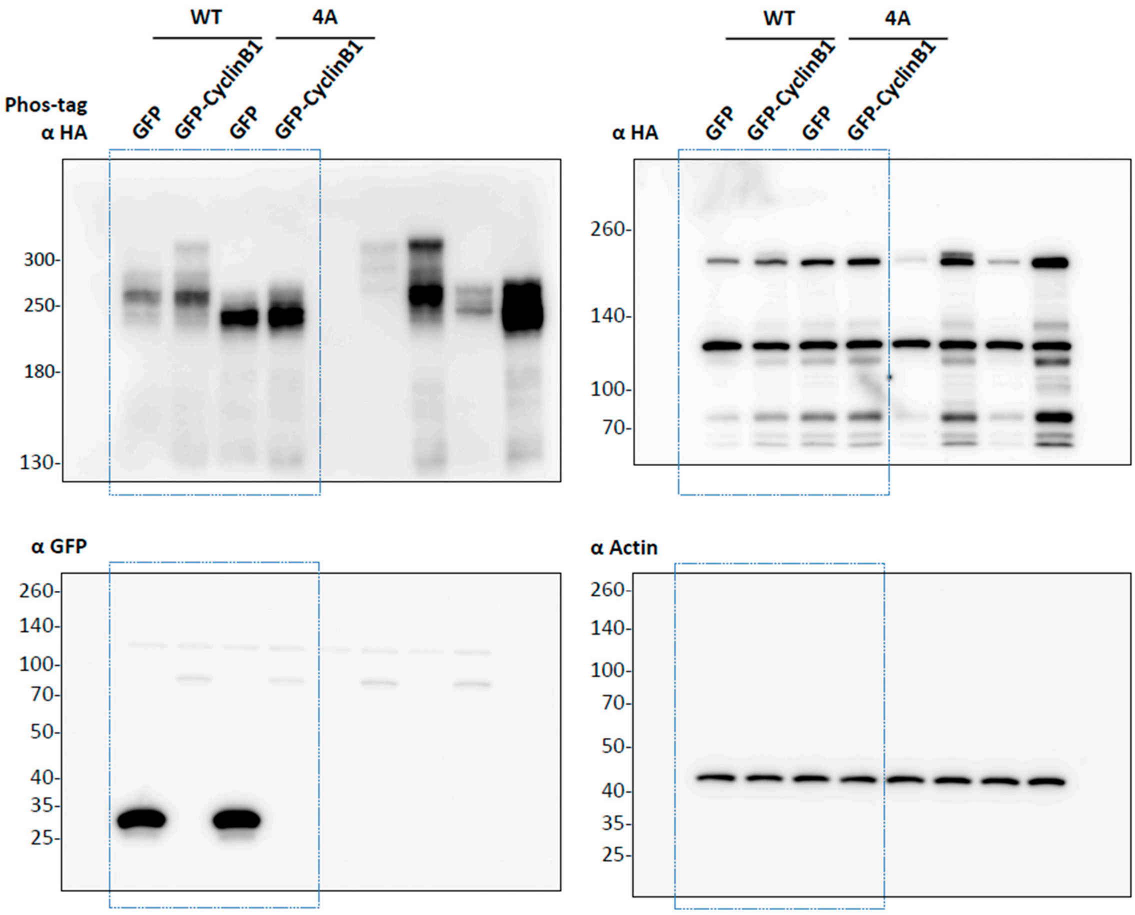
WT
4A
Phos-tag
α HA
GFP
GFP-CyclinB1
GFP
GFP-CyclinB1
300-
250-
180-
130-
WT
4A
α HA
GFP
GFP-CyclinB1
GFP
GFP-CyclinB1
260-
140-
100-
70-
α GFP
260-
140-
100-
70-
50-
40-
35-
25-
α Actin
260-
140-
100-
70-
50-
40-
35-
25-

Figure 4A

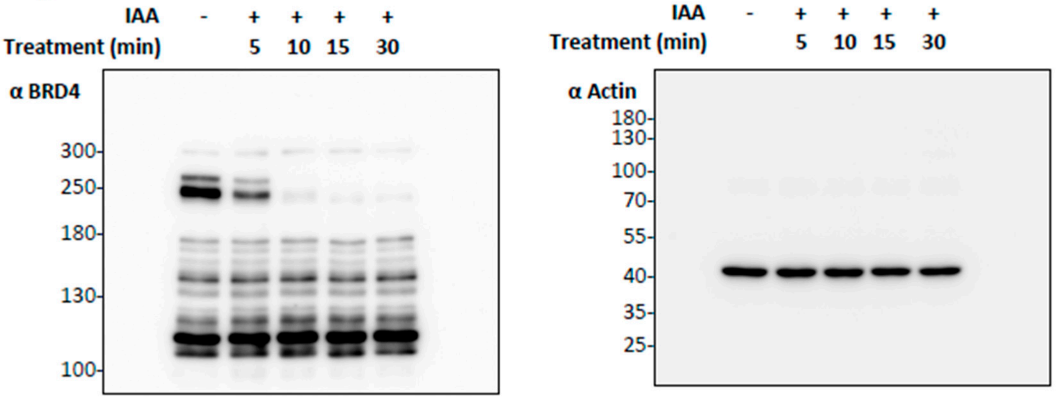

Figure 4B

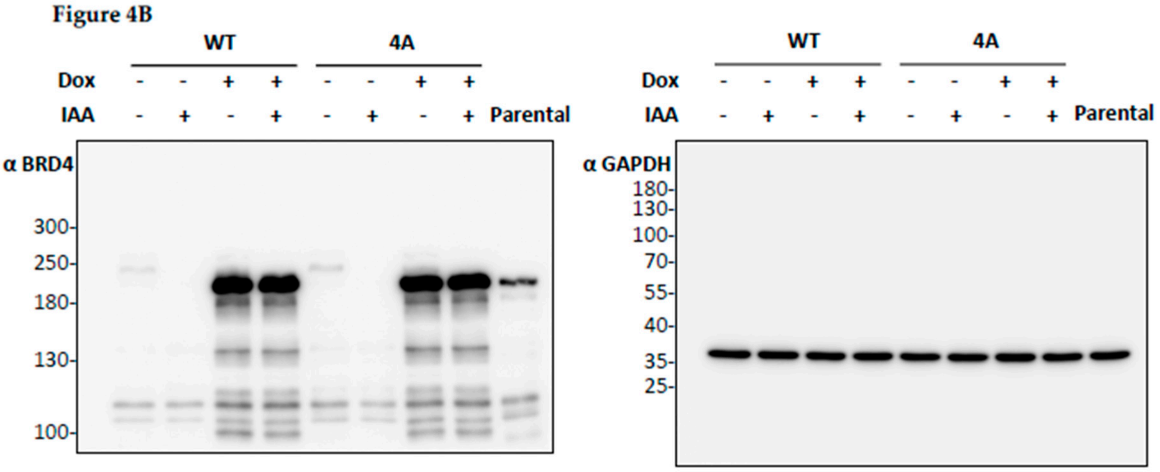

Figure 4C

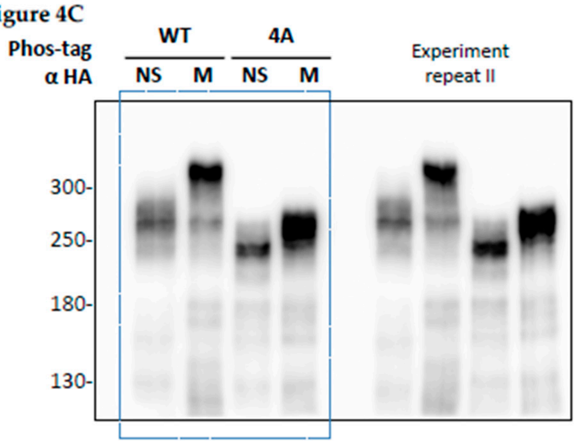

Figure 4D

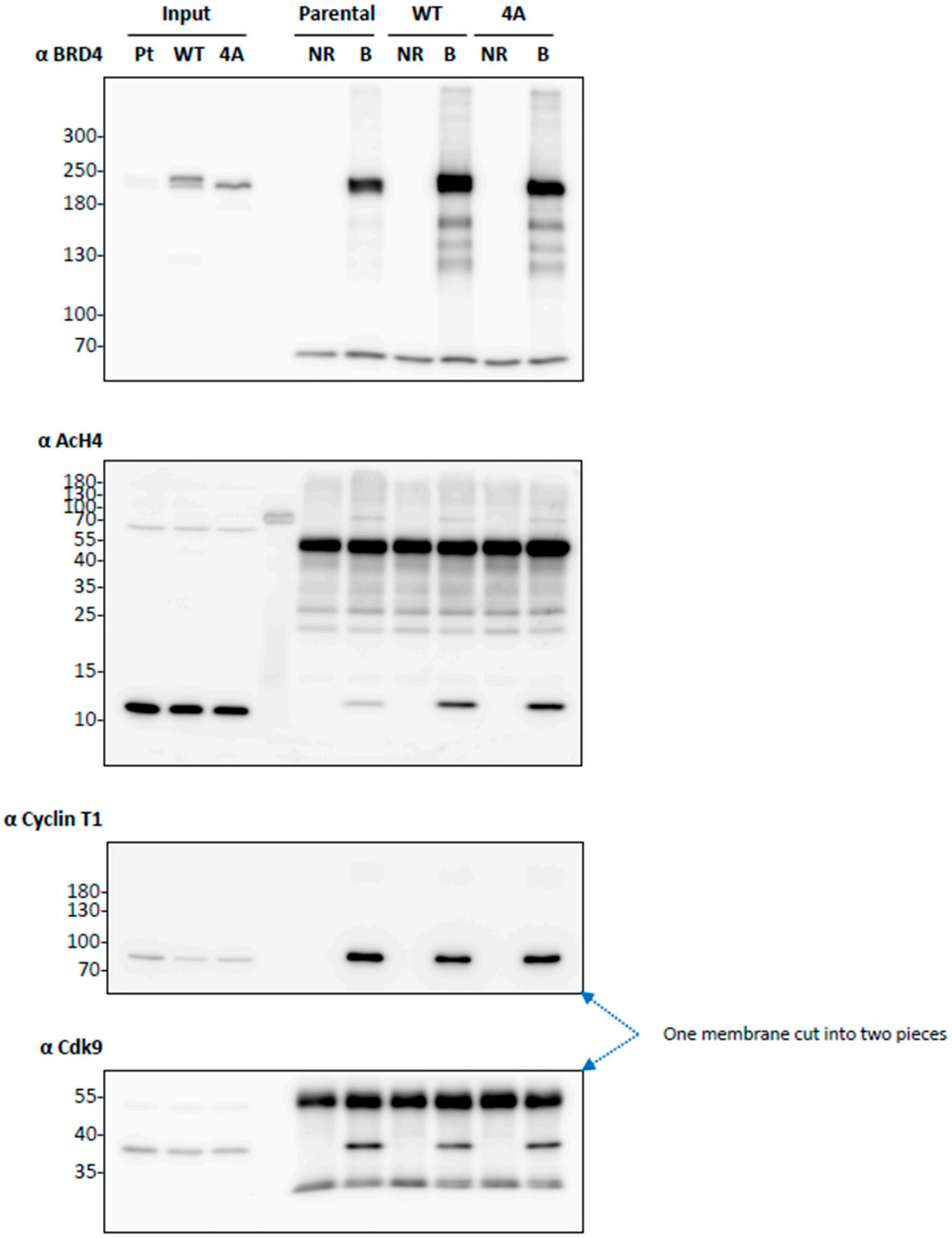
Input
Parental
WT
4A
α BRD4
Pt
WT
4A
NR
B
NR
B
NR
B
300-
250-
180-
130-
100-
70-
α AcH4
180-
130-
100-
70-
55-
40-
35-
25-
15-
10-
α Cyclin T1
180-
130-
100-
70-
α Cdk9
55-
40-
35-
One membrane cut into two pieces

Figure 5C

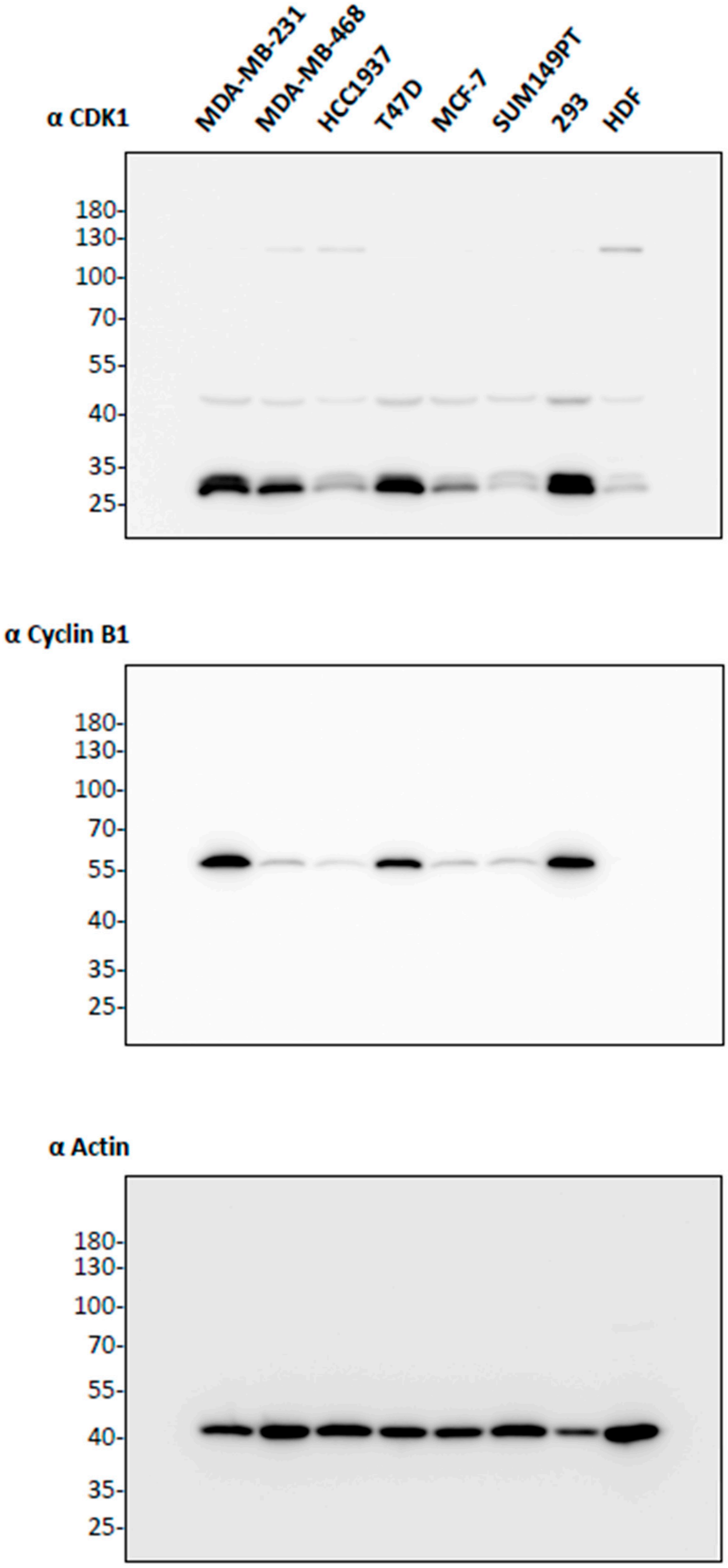

Figure S6. Uncropped immunoblots and gel images.

Figure 1

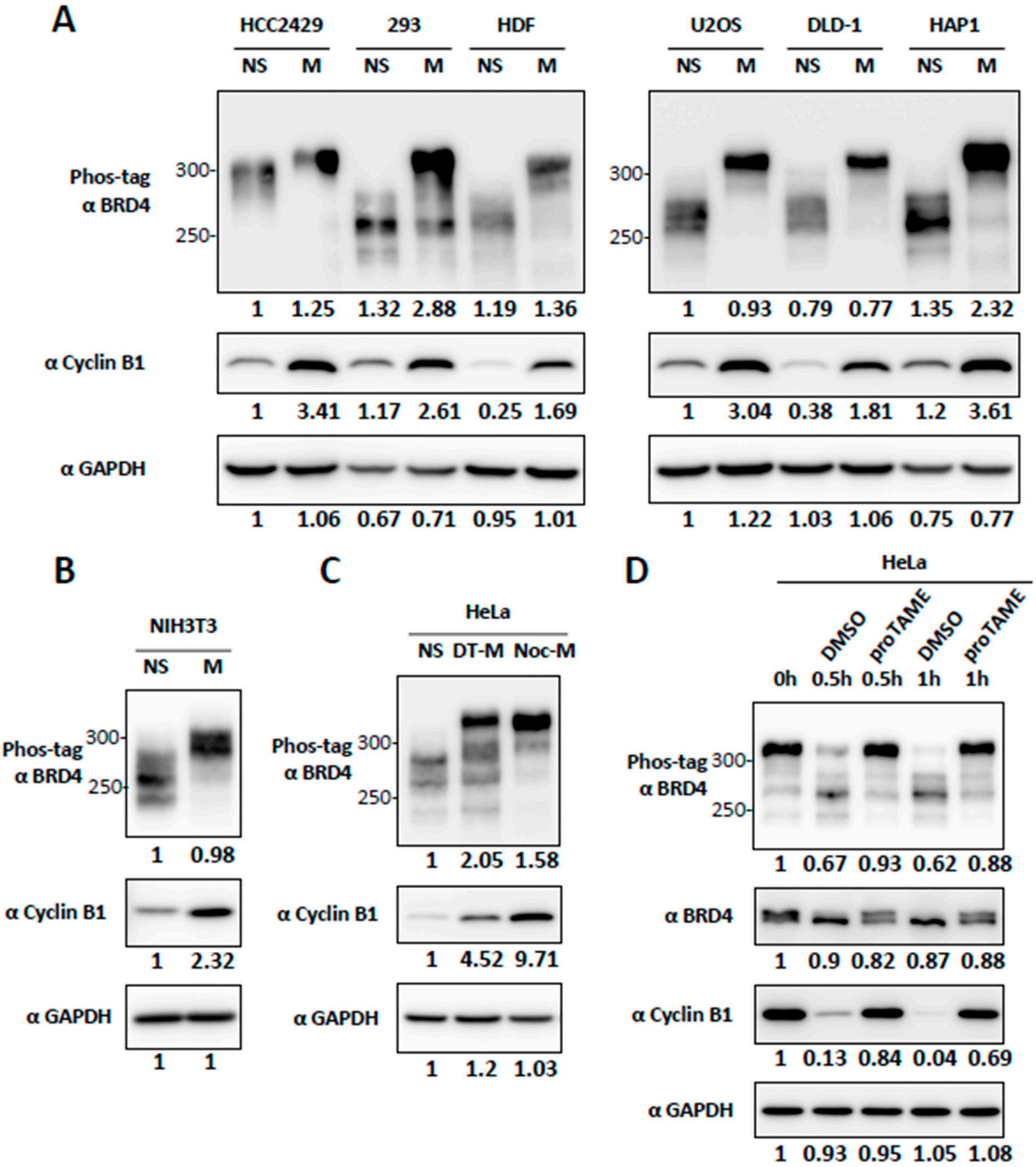
A
HCC2429
293
HDF
U2OS
DLD-1
HAP1
NS M NS M NS M
NS M NS M NS M
Phos-tag
α BRD4
300-
250-
1 1.25 1.32 2.88 1.19 1.36
1 0.93 0.79 0.77 1.35 2.32
α Cyclin B1
1 3.41 1.17 2.61 0.25 1.69
1 3.04 0.38 1.81 1.2 3.61
α GAPDH
1 1.06 0.67 0.71 0.95 1.01
1 1.22 1.03 1.06 0.75 0.77
B
NIH3T3
NS M
1 0.98
1 2.32
1 1
C
HeLa
NS DT-M Noc-M
1 2.05 1.58
1 4.52 9.71
1 1.2 1.03
D
HeLa
DMSO proTAME DMSO proTAME
0h 0.5h 0.5h 1h 1h
1 0.67 0.93 0.62 0.88
α BRD4
1 0.9 0.82 0.87 0.88
1 0.13 0.84 0.04 0.69
1 0.93 0.95 1.05 1.08

Figure 2

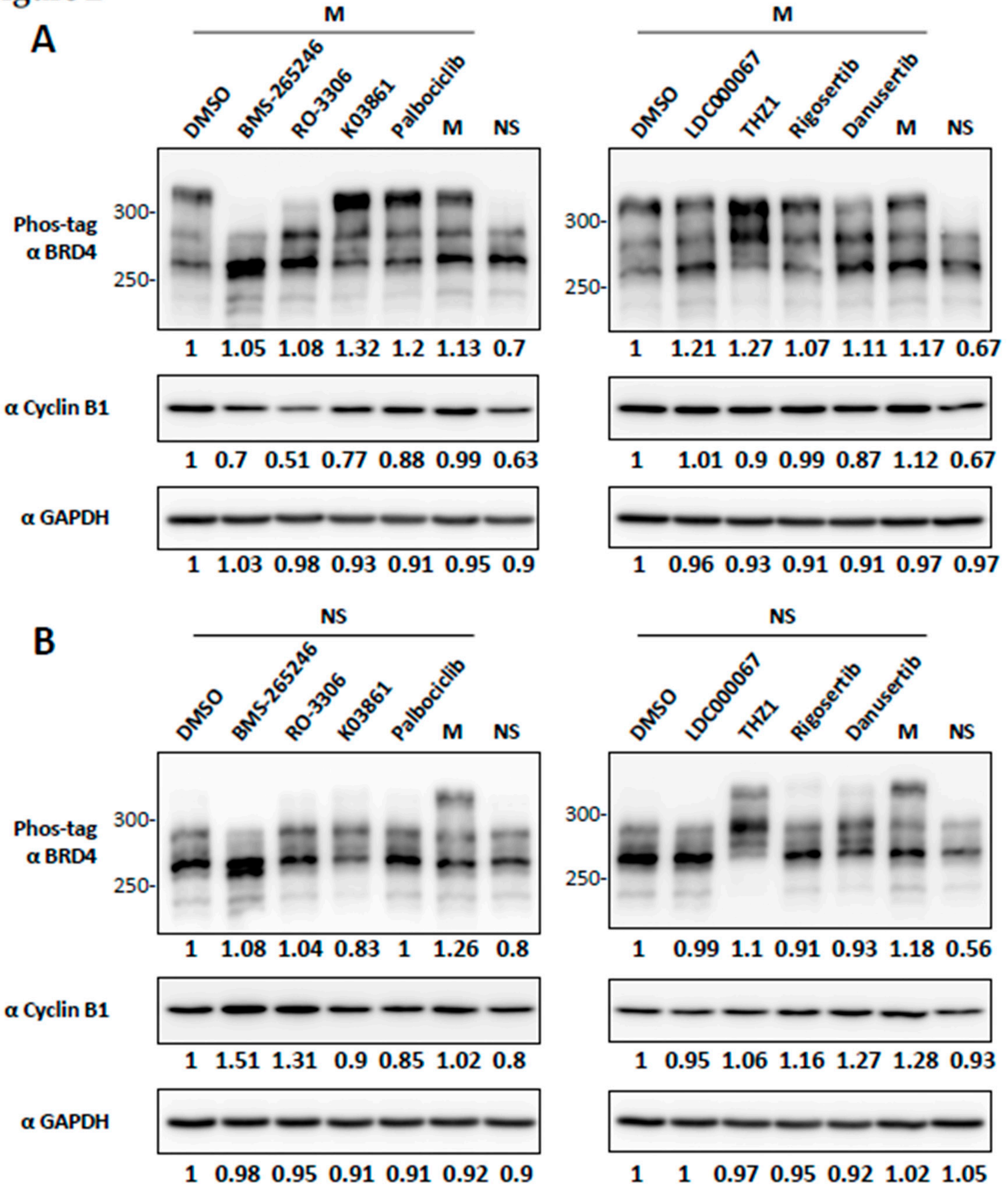
A
M
DMSO
BMS-265246
RO-3306
K03861
Palbociclib
M
NS
DMSO
LDC000067
THZ1
Rigosertib
Danusertib
M
NS
Phos-tag α BRD4
300-
250-
1 1.05 1.08 1.32 1.2 1.13 0.7
1 1.21 1.27 1.07 1.11 1.17 0.67
α Cyclin B1
1 0.7 0.51 0.77 0.88 0.99 0.63
1 1.01 0.9 0.99 0.87 1.12 0.67
α GAPDH
1 1.03 0.98 0.93 0.91 0.95 0.9
1 0.96 0.93 0.91 0.91 0.97 0.97
B
NS
1 1.08 1.04 0.83 1 1.26 0.8
1 0.99 1.1 0.91 0.93 1.18 0.56
1 1.51 1.31 0.9 0.85 1.02 0.8
1 0.95 1.06 1.16 1.27 1.28 0.93
1 0.98 0.95 0.91 0.91 0.92 0.9
1 1 0.97 0.95 0.92 1.02 1.05

Figure 3

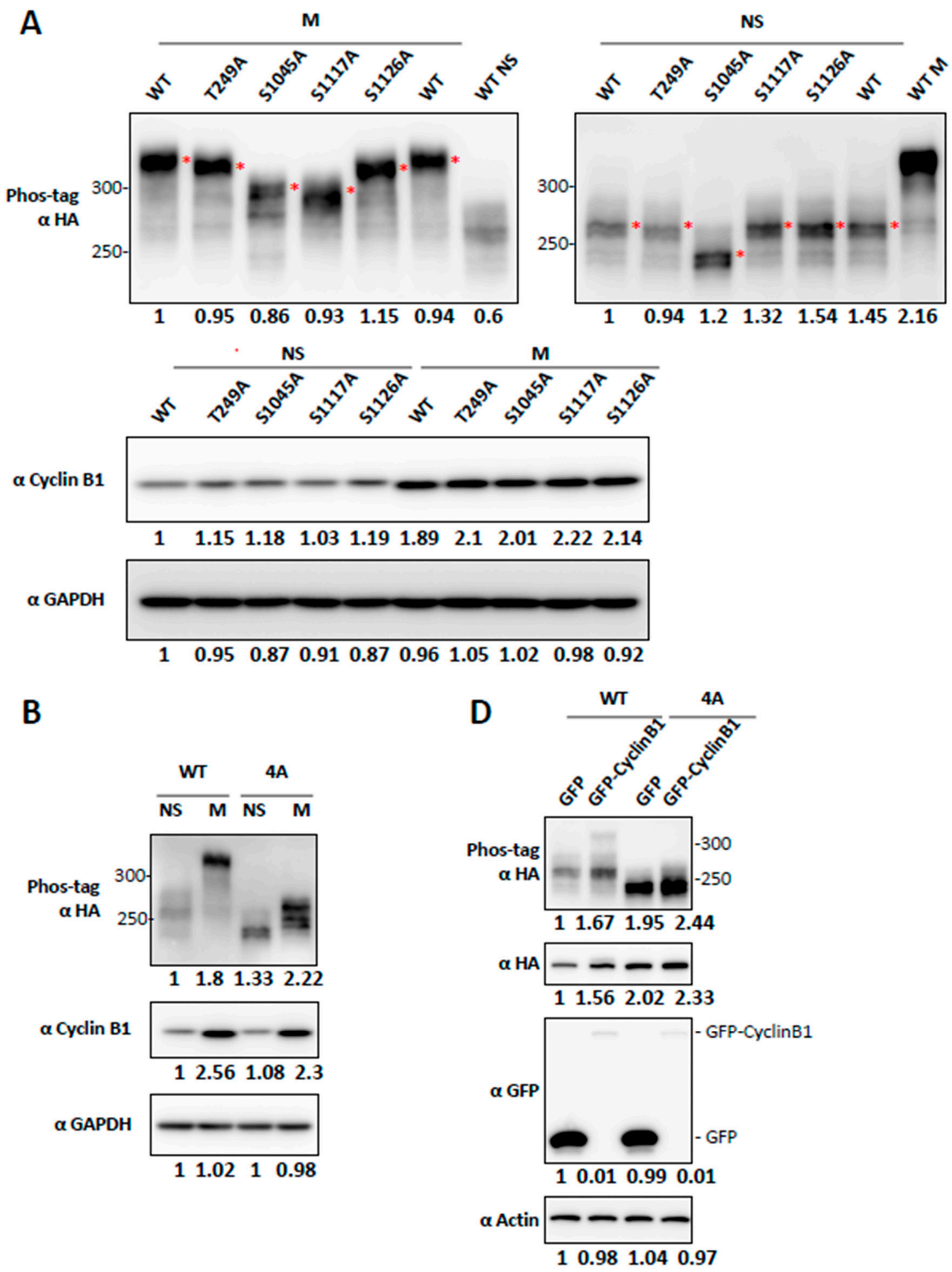
A
M
WT
T249A
S1045A
S1117A
S1126A
WT
WT NS
NS
WT
T249A
S1045A
S1117A
S1126A
WT
WT M
Phos-tag
α HA
300-
250-
1 0.95 0.86 0.93 1.15 0.94 0.6
1 0.94 1.2 1.32 1.54 1.45 2.16
NS
M
WT T249A S1045A S1117A S1126A WT T249A S1045A S1117A S1126A
α Cyclin B1
1 1.15 1.18 1.03 1.19 1.89 2.1 2.01 2.22 2.14
α GAPDH
1 0.95 0.87 0.91 0.87 0.96 1.05 1.02 0.98 0.92
B
WT
4A
NS M NS M
Phos-tag
α HA
300-
250-
1 1.8 1.33 2.22
α Cyclin B1
1 2.56 1.08 2.3
α GAPDH
1 1.02 1 0.98
D
WT
4A
GFP
GFP-CyclinB1
GFP
GFP-CyclinB1
Phos-tag
α HA
-300
-250
1 1.67 1.95 2.44
α HA
1 1.56 2.02 2.33
α GFP
- GFP-CyclinB1
- GFP
1 0.01 0.99 0.01
α Actin
1 0.98 1.04 0.97

Figure 4

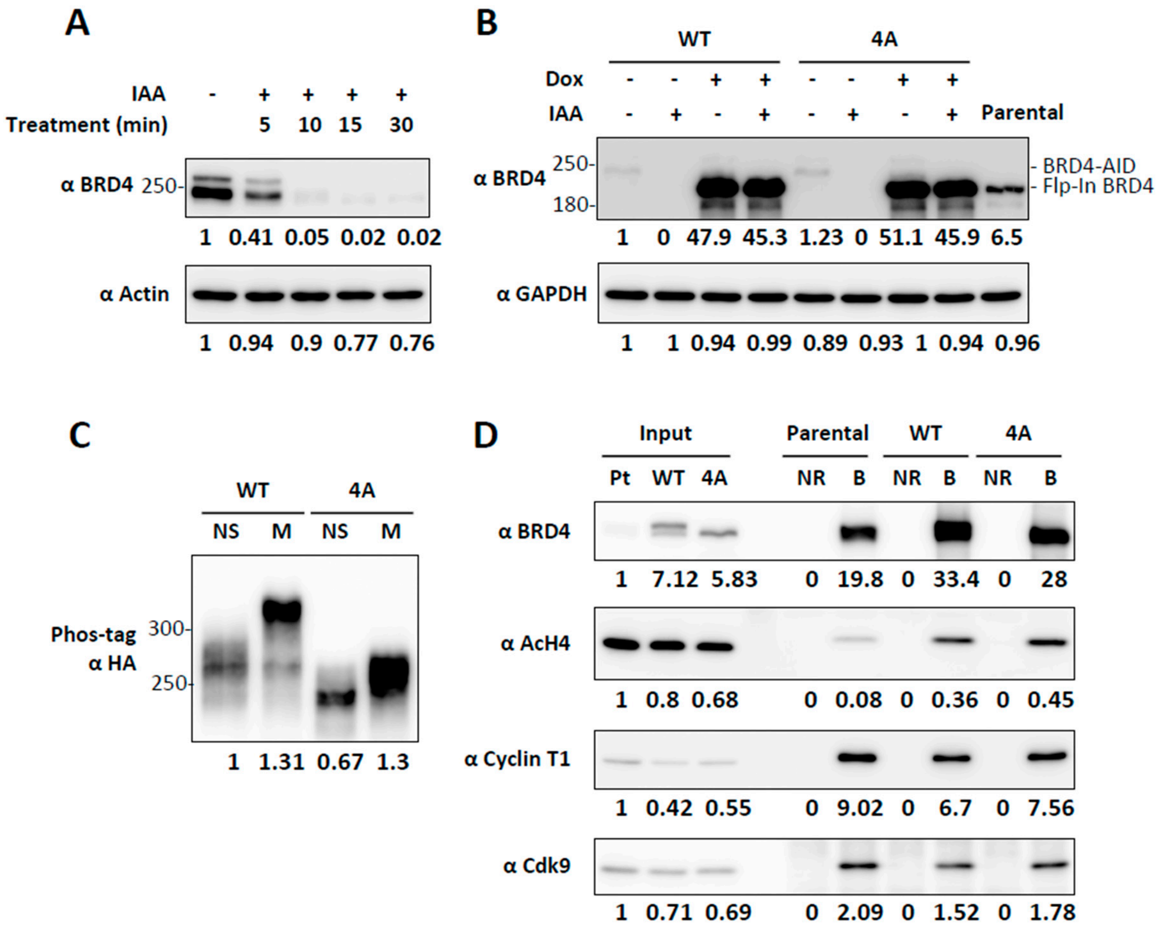

Figure 5

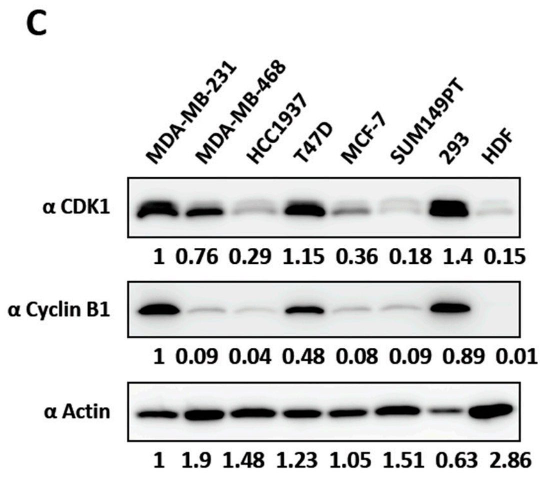

Figure S7. Densitometry reading data.

© 2020 by the authors. Licensee MDPI, Basel, Switzerland. This article is an open access article distributed under the terms and conditions of the Creative Commons Attribution (CC BY) license (http://creativecommons.org/licenses/by/4.0/).
